# Towards the simulation of large scale protein–ligand interactions on NISQ-era quantum computers

**DOI:** 10.1039/d1sc05691c

**Published:** 2022-01-17

**Authors:** Fionn D. Malone, Robert M. Parrish, Alicia R. Welden, Thomas Fox, Matthias Degroote, Elica Kyoseva, Nikolaj Moll, Raffaele Santagati, Michael Streif

**Affiliations:** QC Ware Corporation Palo Alto CA 94301 USA rob.parrish@qcware.com; Medicinal Chemistry, Boehringer Ingelheim Pharma GmbH & Co. KG Birkendorfer Straße 65 88397 Biberach an der Riß Germany; Quantum Lab, Boehringer Ingelheim 55218 Ingelheim am Rhein Germany nikolaj.moll@boehringer-ingelheim.com

## Abstract

We explore the use of symmetry-adapted perturbation theory (SAPT) as a simple and efficient means to compute interaction energies between large molecular systems with a hybrid method combining NISQ-era quantum and classical computers. From the one- and two-particle reduced density matrices of the monomer wavefunctions obtained by the variational quantum eigensolver (VQE), we compute SAPT contributions to the interaction energy [SAPT(VQE)]. At first order, this energy yields the electrostatic and exchange contributions for non-covalently bound systems. We empirically find from ideal statevector simulations that the SAPT(VQE) interaction energy components display orders of magnitude lower absolute errors than the corresponding VQE total energies. Therefore, even with coarsely optimized low-depth VQE wavefunctions, we still obtain sub kcal mol^−1^ accuracy in the SAPT interaction energies. In SAPT(VQE), the quantum requirements, such as qubit count and circuit depth, are lowered by performing computations on the separate molecular systems. Furthermore, active spaces allow for large systems containing thousands of orbitals to be reduced to a small enough orbital set to perform the quantum portions of the computations. We benchmark SAPT(VQE) (with the VQE component simulated by ideal statevector simulators) against a handful of small multi-reference dimer systems and the iron center containing human cancer-relevant protein lysine-specific demethylase 5 (KDM5A).

## Abstract 2

NISQ-era quantum and GPU-accelerated classical computers.

## Graphical abstract

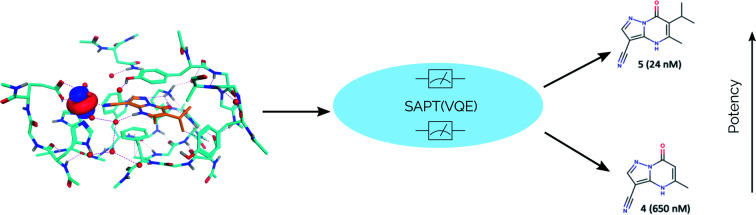

Quantum chemistry has emerged as one of the most promising areas where a practical quantum advantage from near term quantum computers could be demonstrated.^1–3^ Identifying industrially relevant applications that can practically benefit from quantum simulations is, however, a complicated task.^4,5^ On the one hand, existing classical algorithms have benefited from decades of development, benchmarking and optimization so demonstrating a computational advantage over these is challenging given limitations on current quantum hardware.^6–8^ On the other hand, current noisy intermediate-scale quantum (NISQ) hardware^9^ suffers from relatively poor gate fidelity so that the resulting physical properties can often be biased and far from exact without error mitigation.^10^ Thus it is important to design quantum algorithms that minimize the quantum resources required.

Coupled with these challenges is the problem of finding an industrially relevant application that can benefit from a quantum computer in the first place.^11^ One such area that has been suggested as possibly benefiting from quantum computing is computer aided drug design (CADD).^12^ CADD has a long history and has many components ranging from high-level optimization problems such as structure search and conformational sampling down to low-level quantum mechanical problems such as computing protein–ligand interaction energies, all of which could potentially benefit from a large scale quantum computer.^13,14^ In this work we will focus on this final problem, namely computing the interaction energy and properties of large scale protein–ligand systems by approximately solving the electronic structure problem.

To date, most quantum algorithms aimed at solving the electronic structure problem directly are general and have mostly been applied to small molecules in small basis sets. Of course, applications are limited by current quantum resources so that reaching chemical accuracy, defined as calculating energy differences in the complete basis set limit to within 1 kcal mol^−1^ accuracy, is difficult to achieve. Nevertheless, relatively minor attention has been paid to the entire workflow required to solve an industrially relevant problem in drug design. This often includes highly tailored approaches that require classical pre- and post-processing, molecular dynamics and structure relaxation, active space selection and finally computation of interaction energies and related quantities all for systems containing hundreds or thousands of atoms. Thus, it is important to isolate potential application areas now and codify these workflows with quantum algorithms in mind as the pace of hardware improvement accelerates.

Conceptually, computing the interaction energy of a protein–ligand system is a straightforward task. One computes the ground state energy of the dimer and monomer systems separately and subtracts the two to determine the interaction energy. There are a number of issues with this approach, particularly when a quantum computer is involved. First, in finite Gaussian basis sets typically employed in quantum chemical computations, one has to account for basis set superposition error (BSSE) using the counterpoise correction.^15^ This unnecessarily increases the qubit count requirements for the individual monomers and can potentially lead to convergence issues for hybrid quantum–classical algorithms like the variational quantum eigensolver (VQE).^16,17^ A more concerning problem for NISQ computers is resolving total energies of individual monomers (typically on the order of 1000s of kcal mol^−1^) to sufficient precision to subtractively resolve binding energies which are typically on the order of 5 kcal mol^−1^. This is a major issue for NISQ approaches which typically evaluate total energy expectation values statistically, carrying very high measurement cost penalty for high-precision expectation values. This is also an increasingly challenging problem for heuristic algorithms like the VQE which would require very deep circuits with thousands of parameters to achieve the required precision (note that precision and accuracy are the same concern in subtracting total energies unless strict relative error cancellation can be ensured, which is not clear with methods like VQE). This may be practically impossible with the current general algorithms and available hardware although we note alternatives to the VQE may help to overcome this issue.^18^

In this work we propose using symmetry adapted perturbation theory (SAPT)^19,20^ to directly compute the interaction energy through direct expectation values rather than differences, which overcomes some of these problems. Firstly, in principle SAPT does not suffer from BSSE as it directly computes the interaction energy as a perturbation series in the intermolecular potential (note that SAPT still suffers from basis set incompleteness error, as with all second-quantized methods). Secondly, monomer-centered basis sets can be used which can afford additional savings if the geometry of the monomers is fixed across the dissociation path, potentially reducing the number of different quantum computations that have to be performed. Moreover, as SAPT directly computes the interaction energy as a sum of expectation values (rather than differences of large expectation values), it often exhibits favorable error cancellation for errors inherent to the chosen wavefunction ansatz. We show that this observation can significantly reduce the resource requirements for circuit depth with only very coarse VQE wavefunctions required for sub kcal mol^−1^ accuracy in the interaction energy components. Finally, SAPT offers a physically motivated breakdown of the interaction energy components into electrostatic, exchange, induction and dispersion contributions which can offer valuable insight for medicinal chemists when designing protein inhibitors.^21^

Beyond suggesting SAPT as a useful approach for NISQ quantum computers we outline an efficient active space formulation of SAPT that can be applied for protein–ligand interactions for systems containing heavy metal centers and thousands of atoms. Key to this implementation is the GPU accelerated classical pre- and post-processing steps which practically help to run such simulations.^22^ We will largely focus on the accurate qualitative description of systems with strong multi-reference character that can not easily be described by classical approaches and thus could offer a more transparent demonstration of a practical quantum advantage. In this paper we will only consider the first order contributions to the exchange energy leaving the second order terms, which require solving electronic response, for future work.

We begin by outlining in detail the active space formulation for first-order SAPT that can be coupled to any quantum simulation that can produce one- and two-particle reduced density matrices. Next we discuss the VQE ansatz used in this work, although the SAPT method itself is largely independent of the way in which the ground state properties are computed. Finally we benchmark our method using ideal quantum simulators and demonstrate a significant reduction in error found when poorly converged VQE wavefunctions are used for model multi-reference systems and for the human cancer relevant^23^ lysine-specific demethylase 5 (KDM5A) protein with different ligand substitutions.

## I. Methods

In this section we will describe the classical implementation of the density matrix formulation of SAPT followed by how this implementation can be efficiently adapted for NISQ devices.

### A Notation

In this work we consider the interaction of two monomers A and B and will use the following notation for different real orbital types belonging to A and/or B.

• *μ*/*ν* – nonorthogonal atomic spatial orbital basis indices. Note that these could conceptually be either monomer-centered of dimer-centered bases as far as the theory is concerned. Unless otherwise noted, we use monomer-centered bases for all atomic spatial orbital bases encountered in practical test cases in this work.

• *p*/*q* – orthogonal molecular spatial orbital basis indices.

• *i*/*j* – orthogonal occupied spatial orbital basis indices.

• *t*/*u* – orthogonal active spatial orbital basis indices.

• *a*/*b* – orthogonal virtual spatial orbital basis indices.

Repeated indices within a monomer will be denoted with primes, *e.g.*, *p*, *p*′, *p*′′, *p*′′′. Summation over repeated indices is assumed throughout. When dealing with spin-orbital quantities, we use the context specific notation of an “unbarred” orbital index to denote *α* and a “barred” orbital index to denote *β*, *i.e.*, *p*^†^ is an *α* spin-orbital creation operator on spatial orbital index *p*, while *p̄*^†^ is a *β* spin-orbital creation operator on spatial orbital index *p*.

### B Symmetry adapted perturbation theory

Traditionally, the interaction energy between two monomers, *E*_int_, is calculated in the supermolecular approach as

1 *E*_int_ = *E*_AB_ − *E*_A_ − *E*_B_,

where *E*_AB_ is the ground state energy of the combined system and *E*_A/B_ are the energies of the individual fragments, evaluated at the frozen geometry of the dimer system (*i.e.*, no deformation energy contributions). In contrast, in SAPT, *E*_int_ is instead evaluated through a perturbation series in the intermonomer interaction potential2
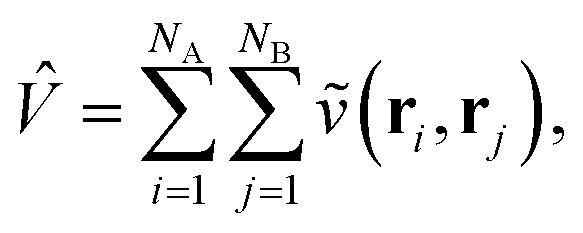
where *N*_A/B_ is the number of electrons in monomer A or B, and3
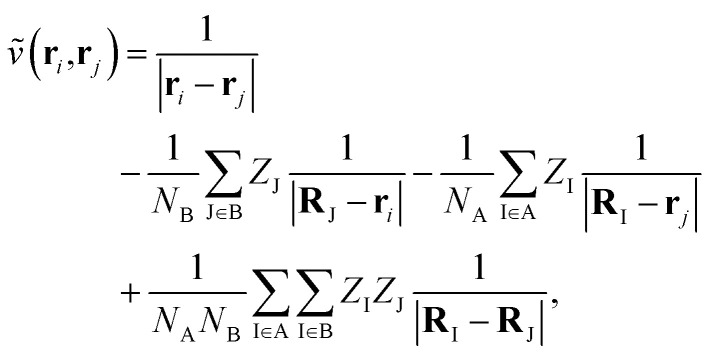
describes the interaction of the electrons and nuclei in monomer A with the electrons and nuclei in monomer B (and *vice versa*). Symmetrized Rayleigh–Schrödinger perturbation theory yields a perturbation series for the interaction energy

4 *E*_int_ = *E*^(1)^_pol_ + *E*^(1)^_exch_ + *E*^(2)^_pol_ + *E*^(2)^_exch_ + …

where, *E*^(1)^_pol_ is the electrostatic energy, *E*^(2)^_pol_ is a sum of dispersion and induction energies, while *E*^(*n*)^_exch_ account for exchange interactions.

In order to evaluate the first two terms in the perturbation series in eqn (4) one first needs to solve for the ground state wavefunction, |*Ψ*_A_〉, of the individual monomer Hamiltonian, here given for monomer A5
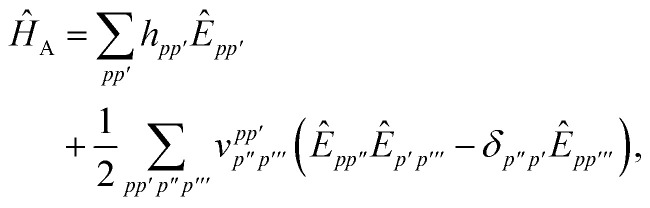
where *h*_*pq*_ and 
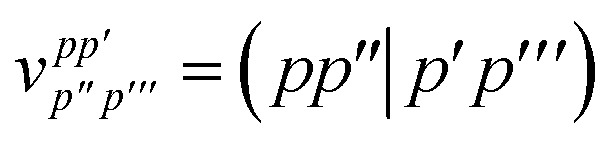
 are the usual one- and two-electron integrals and

6 *Ê*_*pp*′_ = *p*^†^*p*′ + *p̄*^†^*p̄*′,

is the singlet-adapted one-particle substitution operator.

In the absence of the exact ground state monomer wavefunctions, the SAPT interaction energy is traditionally computed as a triple perturbation theory in the intramonomer fluctuation potentials (assuming a Møller–Plesset partitioning of the Hamiltonian). At the lowest order this gives rise to SAPT(HF) (or SAPT0), where each term is evaluated using Hartree–Fock density matrices.^20^ Another popular approach is to use density functional theory SAPT(DFT)^24,25^ to account for intramonomer electron correlation, but this approach is naturally limited by the performance of the chosen density functional.^26^

In this work we will assume that we can determine the exact (or at least very accurate) ground state properties of the individual monomers using a quantum computer. The first order SAPT expressions can then be evaluated using only the ground state unperturbed wavefunctions of the individual monomers, *i.e.*, |*Ψ*_0_〉 = |*Ψ*_A_〉⊗|*Ψ*_B_〉. We will use the density matrix formulation of SAPT^27^ systematized by Korona^28–30^ and recently fully implemented for complete active space self consistent field (CASSCF) wavefunctions by Hapka and *et al.*^26^ This formalism allows for the evaluation of the terms appearing in eqn (4) using just the ground state one- and two-particle reduced density matrices of the monomers with additional response terms for the second order terms. Detailed derivations of the density matrix formulation of SAPT are given elsewhere and here we will focus on the efficient implementation in terms of optimized chemistry primitives on quantum computers.

### C Density matrix formulation of SAPT

The first order polarization energy is the electrostatic repulsion energy of monomer A and B, and is given by

7 *E*^(1)^_pol_ = 〈*Ψ*_A_*Ψ*_B_|*V̂*|*Ψ*_A_*Ψ*_B_〉

8
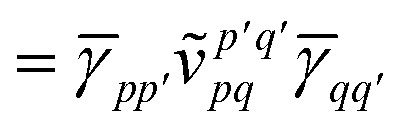
where, *γ̄*_*pp*′_ is the spin-summed one-particle reduced density matrix

9 *γ̄*_*pp*′_ = 〈*Ψ*_A_|*p*^†^*p*′ + *p̄*^†^*p̄*′|*Ψ*_A_〉,

and we have introduced the generalized two-electron repulsion integrals10
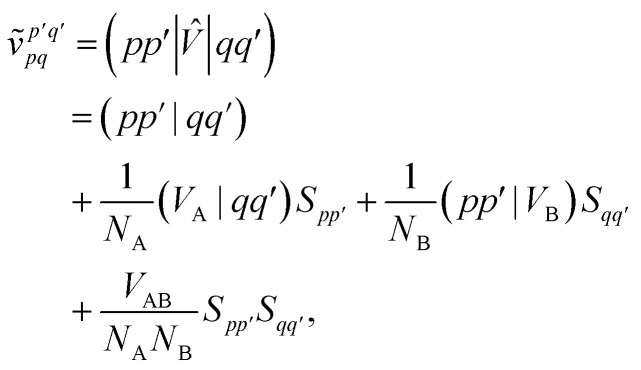
where (*pp*′|*qq*′) is a mixed two-electron electron repulsion integral between orbitals on monomer A and B, *S*_*pp*′_ = (*p*|*p*′) is an overlap integral and (*V*_X_|*qq*′) are matrix elements of the nuclear-attraction potential of monomer X in the orbital basis of the other monomer.

Similarly, the first order exchange energy is given as^27^11
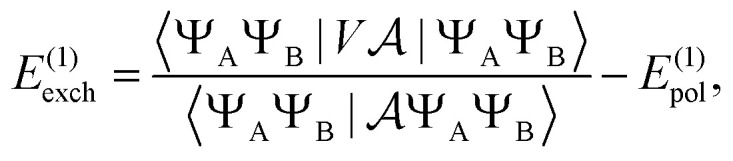
where 
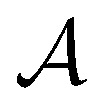
 is the antisymmetrizer operator. Using the *S*^2^ approximation, and the density matrix formulation of SAPT,^27^ it can be shown that eqn (11) can be written as12
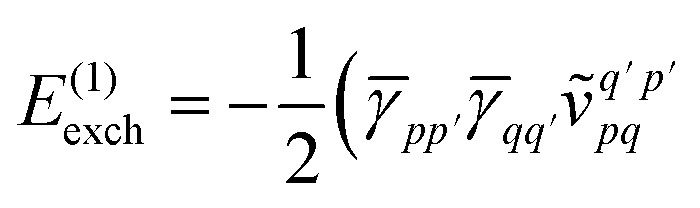
13
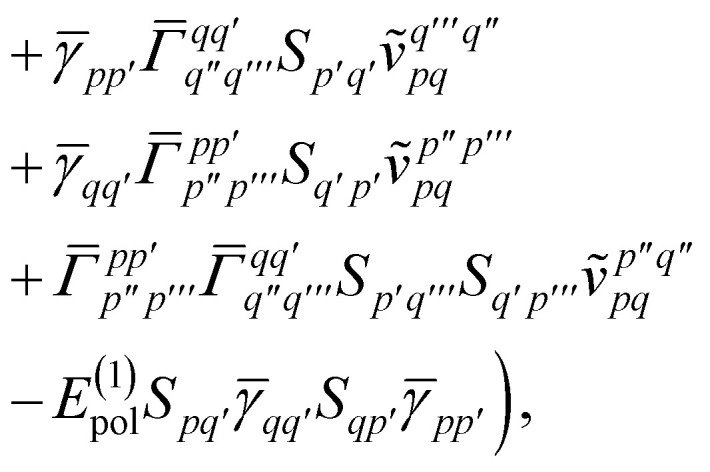

14 = *T*_1_ + *T*_2_ + *T*_3_ + *T*_4_ + *T*_5_

where15
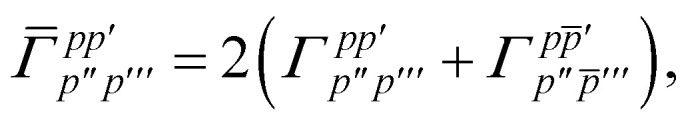
is the spin-summed two-particle reduced density matrix, where throughout this work we assume the monomers have singlet ground states, and16
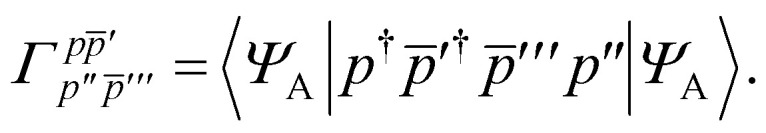


As can be seen from eqn (8) and (14) the first order SAPT expressions require only the one- and two-particle density matrices to be evaluated.

### D Efficient active space implementation

Given that NISQ-era devices are currently limited to tens of qubits (spin-orbitals) we will use an active space approach in order to tackle protein–ligand interactions. We will heavily leverage standard quantum chemistry primitives such as integral driven Coulomb and exchange matrix builds which exploit sparsity^31^ and can efficiently be implemented on GPUs.^32–34^ These considerations are important when simulating hundreds of atoms and thousands of basis functions.

In the active space approach we partition the one-electron orbital set into *N*_c_ core orbitals, *N*_a_ active orbitals and *N*_i_ virtual orbitals. This partitioning gives rise to modified monomer Hamiltonians given by (for example for monomer A)17
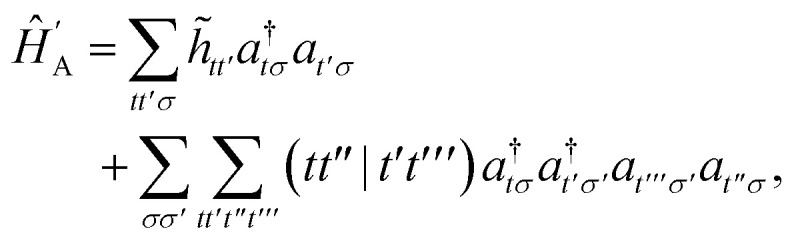
where the modified one-electron integrals *h̃*_*tt*′_ now include core-active space interactions18
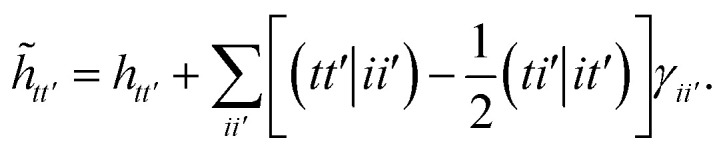


For large scale applications *N*_c_ is typically large (100–1000) and eqn (18) can be efficiently evaluated in the AO basis

19 *h̃*_*μμ′*_ = *h*_*μμ′*_ + 2*J*^c^_*μμ′*_ − *K*^c^_*μμ′*_,

before being transformed to the active space MO basis20
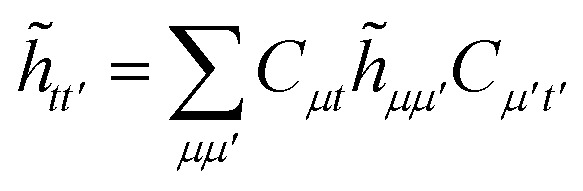
where C are the molecular orbital coefficients. In eqn (19) we have introduced the usual (core) Coulomb and exchange matrices21
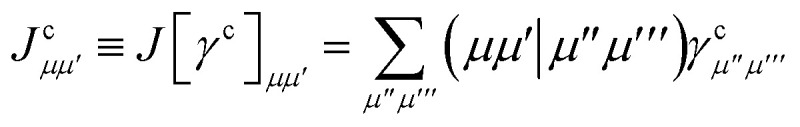
22
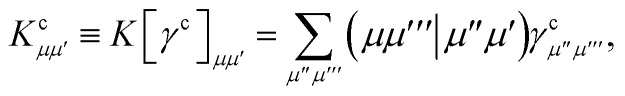
where the core density matrix is given by23
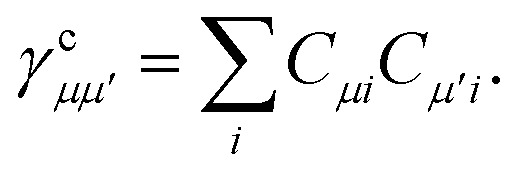


Once the ground state in eqn (17) has been found and the active space one- and two-particle density matrices have been formed the first order SAPT contributions can be calculated as a classical post-processing step which can be implemented efficiently by considering the block structure of the one- and two-particle density matrices. Recall that in the MO basis we have

24 *γ̄*^c^ = *γ̄*_*ii*′_ = 2*δ*_*ii*′_

25 *γ̄*^a^ = *γ̄*_*tt*′_

and26
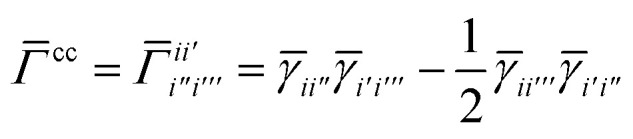
27
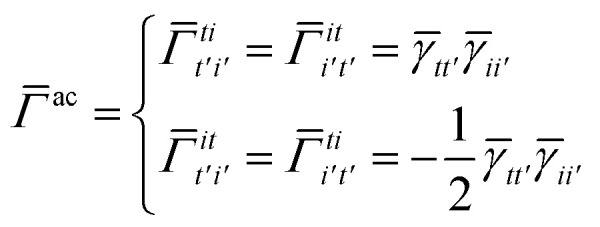
28
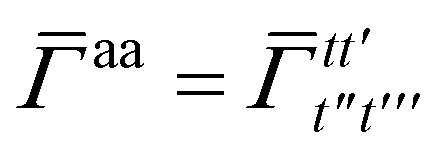
where again *i* and *t* are occupied (core) and active orbital indices respectively. All other blocks of the density matrices are zero.

Not much is gained by exploiting this block structure for first order terms that contain the one-particle density matrix only. Thus, we evaluate them in the AO basis directly as29
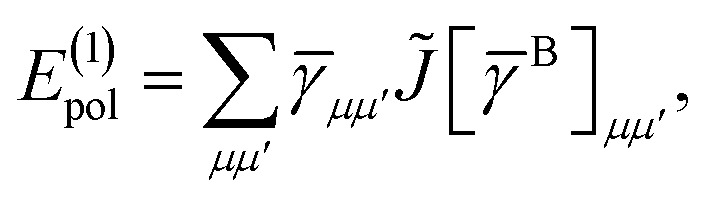
where the generalized Coulomb (*J̃*) and exchange (*K̃*) matrices are analogues of eqn (21) and (22) with the standard electron repulsion integrals replaced with their generalized counterparts (see eqn (10)) and the AO density matrices are given by30
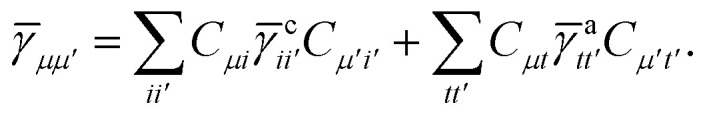


Similarly, the first term in the exchange matrix is evaluated as31
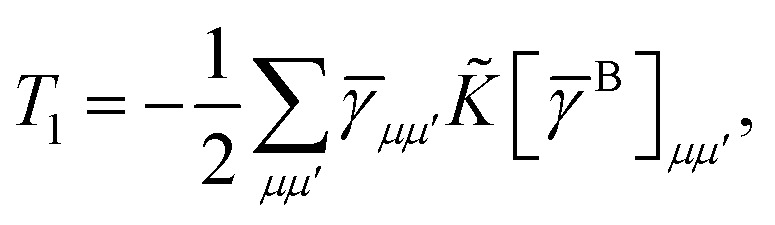
while *T*_5_ is a simple trace of a matrix product. The other three terms are a bit more complicated. For example, for *T*_2_, there will be in total six terms arising from the different combinations of core and active orbitals sets. Inserting the expressions for *γ*^c^ and *Γ*^cc^, *Γ*^ac^ from eqn (28) we have32
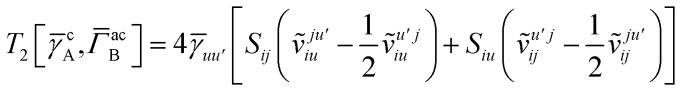


This can be simplified by using the definitions of the generalized Coulomb and exchange matrices defined earlier to be written as33
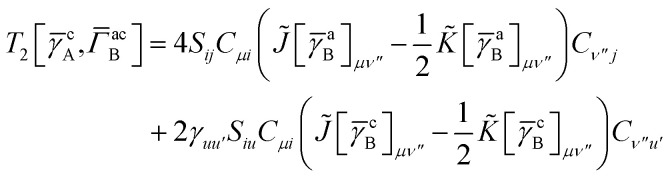


Similarly we have34
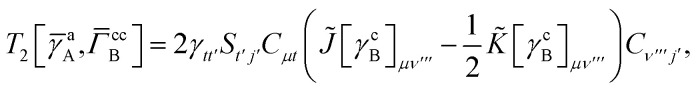
and35
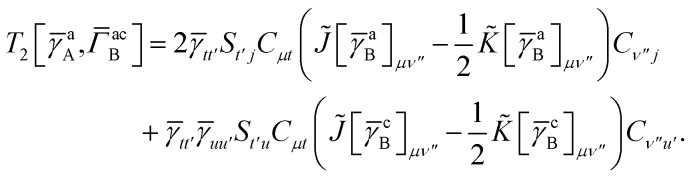


For the core only contribution we find36
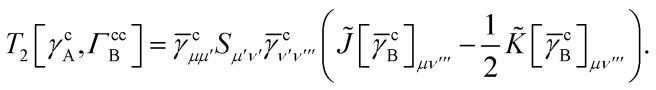


Terms involving *Γ*^aa^ typically cannot be simplified much, *e.g.*,37
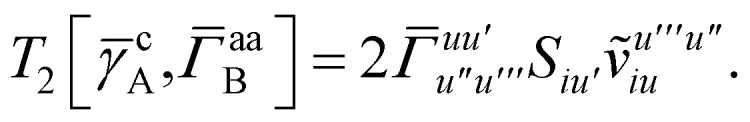


To avoid the formation of the *O*(*N*^3^_a_*N*_c_) generalized electron repulsion integrals it is helpful to note that38
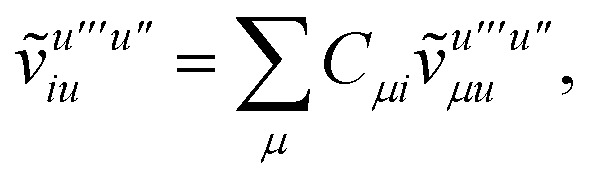
so that39
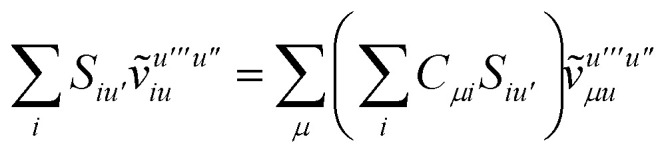
40
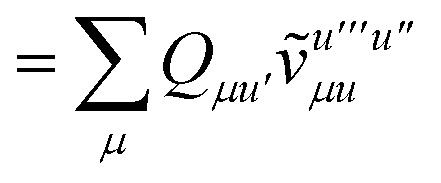
41
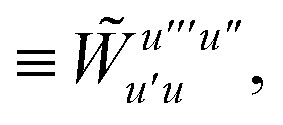
where we have defined42
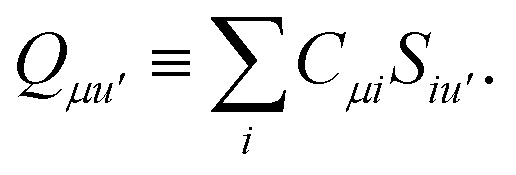


Recall that *S* is an intermolecular overlap integral matrix and will decay to zero as the monomers are separated. This allows us to write43
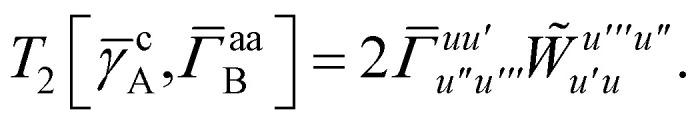


Finally, we have44
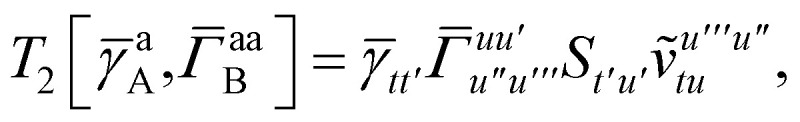
which does not simplify further. Note that the generalized two-electron integrals only need to be constructed for the last two terms and require at most 𝒪(*N*^4^_a_) storage. Although not a concern for the system sizes considered here, further reduction in computational cost and memory can be achieved through density fitting and related approaches.^35^

*T*_3_ is analogous to *T*_2_ while *T*_4_ is quite verbose and contains sixteen terms. Full expressions for these are given in Appendix A. The above expressions are completely general and do not depend on the method used to evaluate the one- and two-particle density matrices.

### E Variational quantum eigensolver

Up to this point we have assumed that the ground state one- and two-particle reduced density matrices of the monomers could be determined by some means. In this subsection we will give further details of the VQE algorithm used in this work. As described, SAPT is essentially a post-processing step that relies only on the availability of the one- and two-particle reduced density matrices. Therefore, it is not tied to any particular quantum algorithm, however, in this work we will focus on using the VQE.

In the SAPT(VQE) approach, one or both of the monomer active space wavefunctions are generated by VQE-type quantum circuits

45 |*Ψ*_VQE_〉 ≡ *Û*_VQE_|*Φ*_I_〉

where |*Φ*_I_〉 is some initial state (typically the Hartree–Fock determinant). Note that with the VQE ansatz adopted for this paper, the active space wavefunction |*Ψ*_VQE_〉 will be taken to be real, and will be a definite eigenfunction of the *N̂*_α_, *N̂*_β_, and *Ŝ*_2_ operators.

In the Jordan–Wigner representation used in this paper, the creation/annihilation operators are defined as,46
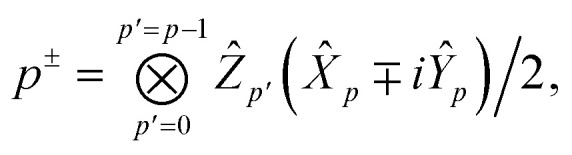
47
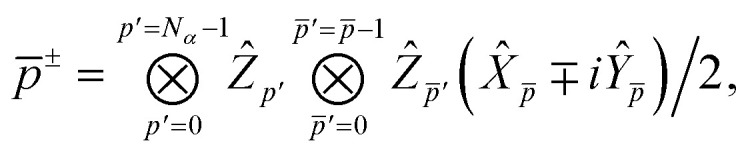
where *p*^+^ = *p*^†^ and *p*^−^ = *p* and we order the Jordan-Wigner strings in α- then β-order and *Ẑ*, *Ŷ* and *X̂* are the usual Pauli operators.

In this work we use a modified version of the unitary cluster Jastrow wavefunction^36^ (*k*-uCJ) which takes the form48
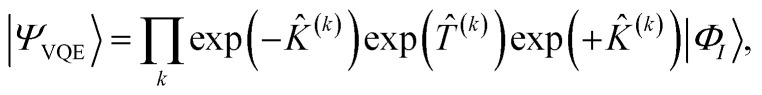
where *K̂*(*k*) and *T̂*(*k*) are one- and two-body operators, and *k* is a parameter that controls the depth of the circuit and as a result its variational freedom. Our modified *k*-uCJ ansatz differs from ref. 36 in the choice of two-body operator and we restrict ourselves to real anti-symmetric matrices. For the one-body rotations we use spin-restricted orbital transformations,49
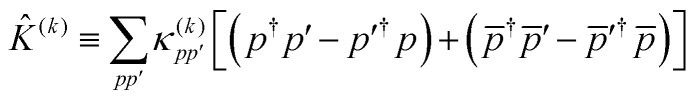
where 
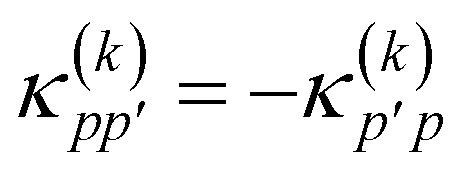
 is a real, antisymmetric *N*_a_ × *N*_a_ matrix of orbital rotation generators. The restricted orbital transformation operator is equivalent to a 1-particle spin-restricted orbital transformation *via*,50
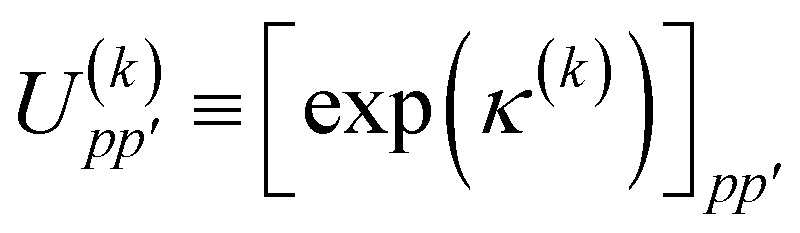


This spin-restricted orbital rotation can be efficiently implemented in quantum circuits *via* a fabric of Givens rotations.^37^

**Fig. 1:**
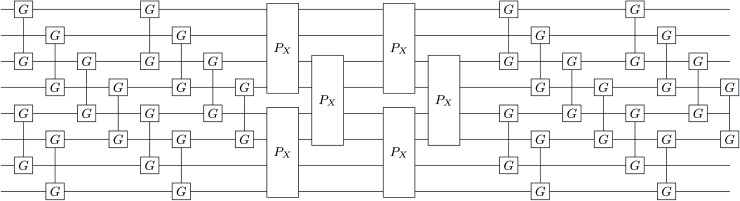
Single layer (*k* = 1) *k*-muCJ VQE entangler circuit used in this work sketched for *M* = 4 spatial orbitals or *N* = 8 qubits. Even (odd) qubits label α (β) spin-orbitals. The circuit consists of a single layer of orbital rotations which are implemented as a series of two-qubit Givens rotations, followed by a layer of diagonal double substitution operators implemented *via* the four-qubit pair-exchange gate given in eqn (52), followed by another layer of orbital rotations. Note that for the spin-restricted ansatz used here the angles in the Givens gates for α and β spin-orbitals are the same.

For the two-particle operator we use a modified diagonal double-substitution operator,51
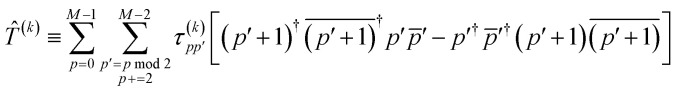
which is similar to the unitary pair coupled-cluster generalized singles and doubles expression (*k*-UpCCGSD).^38^ The summation over the qubit index in eqn (51) is rather verbose, but can be understood as alternating between all pairs of nearest-neighbor spatial orbitals, with alternating 0 (even) and 1 (odd) starting spatial orbital. This pattern of summation indices is visually indicated in the *P*_X_ gates in Fig. 1. Note that this variant of the uCJ ansatz is similar in structure to the quantum number preserving fabric circuit^39^ or the fermionic SWAP network implementation of *k*-UpCCGSD.^40^ However, as our choice is not quite any of these ansatzes, for the remainder of this work we will name it *k*-muCJ for clarity, with the ‘m’ standing for modified. We stress again that the choice of VQE ansatz is largely irrelevant from a SAPT perspective and is not a major point in this paper.

The diagonal doubles operator in eqn (51) can be implemented as a product of four-qubit pair-exchange gates, *P̂*_X_(*θ*), which have the action,52
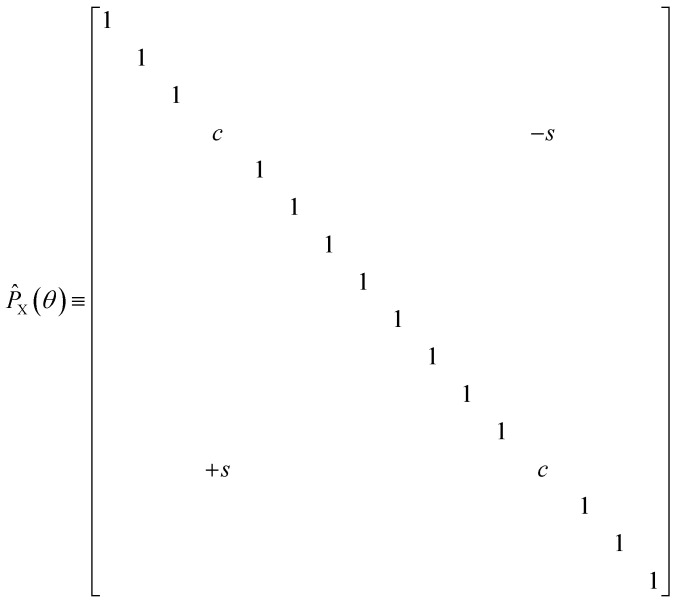
in the four-qubit Hilbert space and in the above we use the shorthand *s* = sin(*θ*) and *c* = cos(*θ*). Note that, this operator implements a partial double-substitution in the closed-shell space of the four-qubit Hilbert space.^39^ The decomposition of eqn (52) into more standard gates is given in ref. 39. An example of one layer of the muCJ circuit ansatz is given in Fig. 1.

With an ansatz of the form of eqn (48) we can write the VQE objective function as

53 *E*_VQE_(*κ*^*k*^_*pq*_,*τ*^*k*^_*pq*_) ≡ 〈*Ψ*_VQE_(*κ*^*k*^_*pq*_,*τ*^*k*^_*pq*_)|*Ĥ*|*Ψ*_VQE_(*κ*^*k*^_*pq*_,*τ*^*k*^_*pq*_)〉

54 = 〈*Φ*_I_|*Û*^†^(*κ*^*k*^_*pq*_,*τ*^*k*^_*pq*_)*ĤÛ*(*κ*^*k*^_*pq*_,*τ*^*k*^_*pq*_)|*Φ*_I_〉.

The VQE algorithm then proceeds in hybrid form by using the quantum computer to evaluate eqn (54) before the variational parameters {*κ*^*k*^_*pq*_,*τ*^*k*^_*pq*_} are updated using a classical optimization algorithm.

To estimate the one- and two-particle reduced density matrices we write the expectation values in eqn (9) and (16) in terms of the Jordan-Wigner strings. The number of measurements scales like 
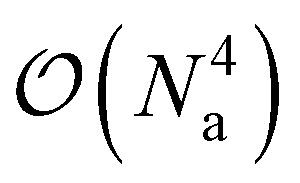
 which can be reduced to an extent by accounting for symmetries in the one- and two-particle density matrices. Although not a focus of this paper, efficiently estimating density matrices is an active area of research and techniques exist to increase the parallelism of the measurements^41^ and for error mitigation.^42^

## II. Results

### A Computational details

**Fig. 2:**
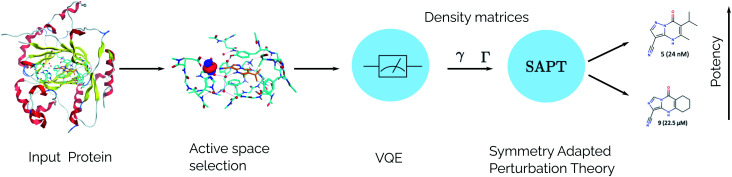
SAPT(VQE) workflow for computing intermolecular interaction energies. In the first stage the input structures for the protein and ligand are provided. Following this, the structures are typically relaxed using classical molecular dynamics and DFT and a model system of the binding site is cutout from the full structure. At this stage an active space for the protein and/or ligand can be determined. Next, a quantum algorithm like the VQE is used to determine the one- and two-particle reduced density matrices which serve as input for SAPT. The SAPT interaction energy is finally computed as a classical post-processing step. Note that only the determination of the one- and two-particle density matrices (*γ* and *Γ* respectively) should be performed on a quantum computer.

For the idealized experiments presented here we use the L-BFGS-B algorithm provided by scipy^43^ to optimize the VQE objective function and used the RHF state as the initial wavefunction. Classical SCF computations and integral generation was performed using Terachem^32–34^ interfaced through Lightspeed.^44^ The GPU accelerated ideal VQE simulations were implemented using the quasar/vulcan codes. Double factorization was used for evaluating the total energy of the VQE ansatz.^45,46^ VMD^47^ was used for visualizing molecular orbitals and molecular structures with the exception of the KDM5A system which used MOE.^48^ A sketch of the workflow is given in Fig. 2.

### B Multi reference benchmarks

To assess the accuracy of SAPT(VQE) we will apply the method to investigate the intermolecular electrostatic and exchange energy to a selection of dimers from the S22 benchmark set.^49^ We will modify these systems to ensure one of the monomers in question has some multi reference character that cannot be well described by SAPT(RHF). Note that previous studies of SAPT(CASSCF) have found little benefit of the method for single-reference problems in the S22 benchmark set,^26^ so we only focus on more challenging multi-reference problems. In all examples we will benchmark our results against classical SAPT(CASCI) that employs the same active space as the SAPT(VQE) computations.

**Fig. 3:**
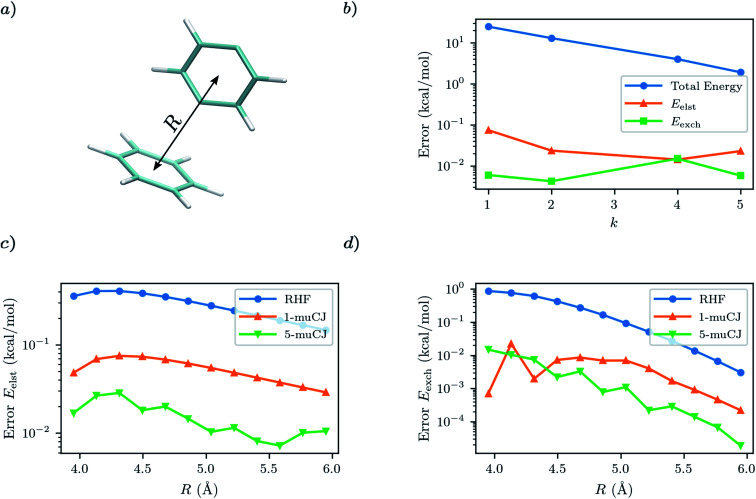
(a) T-shaped configuration of benzene–*p*-benzyne dimer with the center-to-center intermonomer distance labelled as *R*. (b) Absolute error in *p*-benzyne monomer VQE total energy compared to errors in the SAPT(VQE) electrostatic and exchange energies at *R* = 4.45 Å intermonomer separation as a function of the circuit repetition factor *k* in the *k*-muCJ ansatz. (c and d) Absolute errors relative to SAPT(CASCI) in the electrostatic and exchange energies calculated using different monomer wavefunctions for *p*-benzyne as a function of the intermonomer separation.

For our first test case we will investigate a benzene–*p*-benzyne dimer arranged in the T-shape configuration visualized in Fig. 3(a). This is a variation on the classic benzene dimer SAPT benchmark, except *p*-benzyne has a biradical ground state and thus benefits from a multi-reference approach. To describe *p*-benzyne we construct a (6e, 6o) active space from the HOMO-2 to LUMO+2 RHF/cc-pVDZ MOs and treat the benzene monomer at the RHF level of theory. Previous results suggest that *p*-benzyne represents a challenge for VQE with the number of parameters required to reach chemical accuracy being roughly half the number of configuration state functions in the exact solution.^39^ Thus, it represents an interesting test case to see how errors from VQE propagate through to the resultant SAPT energy components.

As can be seen from Fig. 3, the *p*-benzyne molecule does represent a challenge for the *k*-muCJ ansatz, with a *k* > 5 required to reach an error in the total energy below 1 kcal mol^−1^. In contrast we see that the errors in the SAPT(VQE) electrostatic and exchange energies are 2–4 orders of magnitude smaller than the corresponding VQE total energy and that a shallow depth *k* = 1 VQE ansatz would be sufficient for sub kcal mol^−1^ accuracy in these terms. We see this holds across the dissociation curve, which is expected as the VQE solution is fixed when working in a monomer-centered basis set, which is another advantage of SAPT(VQE). It should be noted that the SAPT(RHF) binding energy for this dimer system is on the order of 2 kcal mol^−1^ for this basis set.

**Fig. 4:**
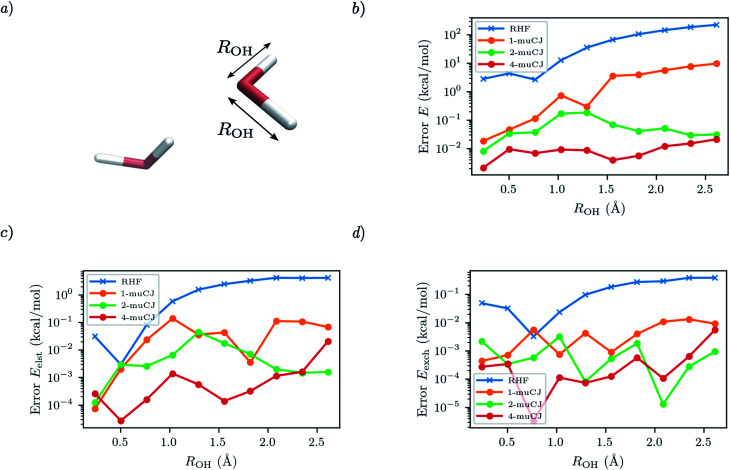
(a) Geometry of the water dimer studied with the OH bond length of single monomer distance labelled as *R*_OH_. The intermonomer separation is fixed throughout. (b) Absolute error in the stretched water monomer VQE total energy as a function of *R*_OH_ for different muCJ repetition factor *k* in the *k*-muCJ ansatz. (c and d) Absolute errors relative to SAPT(CASCI) in the electrostatic and exchange energies calculated as a function of *R*_OH_ for different muCJ repetition factor *k* in the *k*-muCJ ansatz.

For the next test case we investigate the ability of SAPT(VQE) to address intermolecular interactions involving bond dissociation in one of the monomers. We calculate the SAPT electrostatic and exchange energies for two interacting water molecules (displayed in Fig. 4(a)), one in its equilibrium geometry and in the other we symmetrically stretch the two OH bonds towards dissociation. This is again a challenging case for VQE to capture a double bond breaking. We used the 6-31G basis set and chose a (6e, 6o) active space for water from HOMO−2 to LUMO+2. Again, we see in Fig. 4 that RHF qualitatively fails to capture either the total energy of the stretched water monomer or the SAPT energy components of the dimer system. On the other hand, while sizeable errors are present in the VQE total energy (>10 kcal mol^−1^) in the stretched system for *k* = 1, we find sub 0.1 kcal mol^−1^ accuracy in the SAPT energy components.

It is interesting to note that while the quality of the total energy necessarily improves when the circuit repetition factor (*k*) is increased (assuming a local minima is not arrived at during optimization), some non-monotonic behaviour is observed in the individual SAPT energy components. This is probably due to the difficulty in tightly converging the VQE total energy in log scale in these figures.

These two examples provide strong evidence that SAPT(VQE) yields significantly lower absolute errors in the target interaction energy contributions *vs.* those of the corresponding monomer total energies. This helps to reduce the depth of circuits necessary to achieve accurate interaction energy components.

### C Protein–ligand interactions

**Fig. 5:**
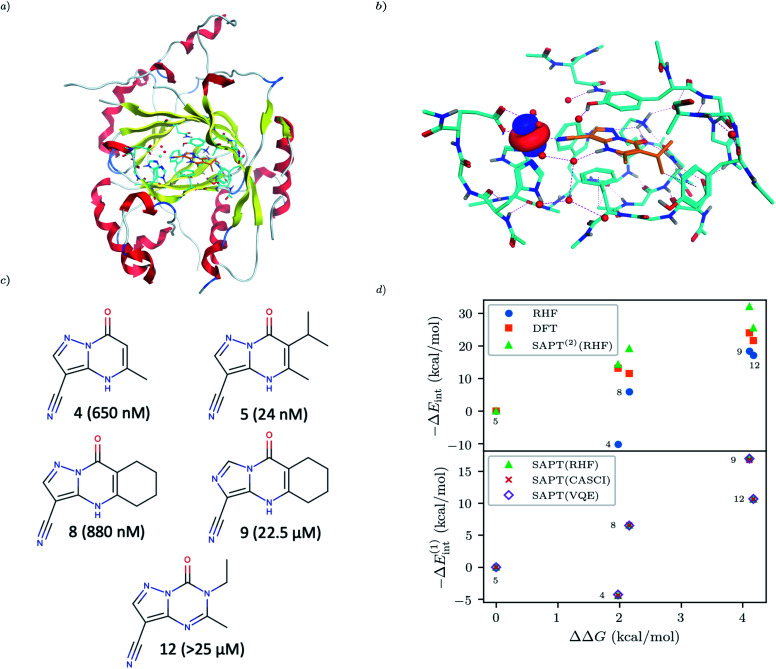
(a) KDM5A protein structure^50,51^ (b) representative minimal active space molecular orbitals produced by the AVAS procedure, for the protein cutout described in the main text with ligand-5 colored as orange (c) 5 KDM5A inhibitors from ref. 52, (d) Upper Panel: Interaction energy differences computed using the super molecular approach (DFT(*ω*B97X-D) and RHF) and SAPT^(2)^(RHF) as a function of the difference in experimental free energies (see main text for definition). Lower Panel: First-order SAPT(VQE), SAPT(CASCI) and SAPT(RHF) first order interaction energy differences as a function of the differences in experimental free energies of binding. Note that Δ*E*_int_(*x*) = *E*_int_(*x*_ref_) − *E*_int_(*x*) so − Δ*E*_int_ will be positive if the ligand is predicted to have a smaller interaction energy (*i.e.* less negative) than the reference ligand (here taken to be ligand-5).

For our final example we examine the ability of our implementation of SAPT(VQE) to tackle large protein–ligand interactions, specifically lysine-specific demethylase 5A (KDM5A) depicted in Fig. 5(a). KDM5A is believed to be relevant for human cancers^23^ and contains a metal center [Fe(ii)] which may pose a challenge for classical electronic structure theory methods. To make the problem tractable, we used a model system of the binding site which was cut out from the full binding domain of KDM5A and focuses on the immediate surrounding of metal ion and ligand. The detailed preparation of the model system is described in the Supporting Information. This final structures contained each 380 atoms and were studied with DFT using a 6-31G basis set and the *ω*B97X-D functional^53^ using the Gaussian software package.^54^ The ligand and all atoms in radius of 4.5 Å of the ligand were further relaxed with the oxygen atoms of the water and the iron atom been keeping fixed. The structures have an electronic size of (1482e, 2214o). The protein–ligand system is visualized in Fig. 5(b). We computed the first order SAPT(VQE) energy contributions for 5 different inhibitors from ref. 52 and shown in Fig. 5(c) to assess how important multi-reference effects were for describing the interaction energy with this protein. We treated each of the ligands at the RHF level and KDM5A using VQE.

To construct a potentially representative active space for KDM5A we used the AVAS procedure,^55^ and first built a relatively large space by including the 3d orbitals from the iron center, the oxygen 2p orbitals from the nearest two water molecules and the 2p orbitals from the neighbouring oxygen atom associated with the glutamic acid, as well as two 2p orbitals from the neighbouring nitrogen atoms from two neighbouring histidines. A representative Fe 3d-like orbital from this procedure is depicted in Fig. 5(b). This leads to an active space size from the AVAS procedure of (36e, 27o), which would require a 54 qubit quantum computer, and is thus outside the reach of current simulators and hardware. To reduce the size of the active space we performed a loosely converged (*ε* = 1 × 10^−4^) selected heat bath configuration interaction computation (SHCI)^56,57^ in this active space. We then constructed a smaller active space using the SHCI natural orbitals (NOs), only keeping those NOs with occupation 0.02 ≤ *n*_i_ ≤ 1.97. This leads to a smaller active space of (8e, 8o), for the low-spin configuration, corresponding to a 16 qubits VQE simulation which is possible using simulators and within reach of current hardware. The natural orbital occupation structure and dominant single reference nature of SHCI ground state suggests that the KDM5A problem will not be challenging for VQE.

In order to connect with experiment we score the ligands based on the difference of their interaction energy relative to a reference ligand

55 Δ*E*_int_(*x*) = *E*_int_(*x*_ref_) − *E*_int_(*x*)

where *x* are the ligand labels from Fig. 5(c) and we choose *x*_ref_ = 5 as the reference point as it is experimentally the most potent ligand from the subset of ligands taken from ref. 52. We then compare this energy difference to differences in the experimental free energy of binding^58^56
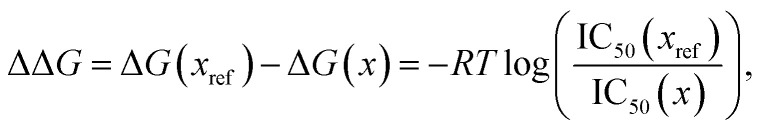
where *R* is the gas constant, the temperature *T* is taken to be room temperature and we took the IC_50_ values from ref. 52. Note that we are comparing two different quantities, namely the theoretical interaction energy difference and the experimental free energy of binding and thus we cannot necessarily expect any quantitative relationship. For example, the interaction energies account for one leg of the thermodynamic cycle while the free energy of binding accounts for the whole cycle. In addition, the number of water molecules surrounding the binding site may depend on the specific ligand being studied, with larger ligands replacing more water molecules. While neither of these effects was considered here it is interesting to see what relationship if any these numbers have to one another.

The results of these computations are shown in Fig. 5(d). To get some insight into the accuracy of existing approaches we first present supermolecular DFT(*ω*B97X-D), RHF and SAPT^(2)^(RHF) the results of which are shown in the upper panel of Fig. 5(d). Note that here SAPT^(2)^(RHF) contains both first- and second-order SAPT energy contributions^22^ and were performed in a dimer-centered basis set, this in contrast to the rest of the results in this paper which only considered the first-order SAPT contributions in a monomer-centered basis. The DFT results are not counterpoise corrected but we found this consideration unimportant for the present system. We find that supermolecular RHF incorrectly predicts ligand-4 to have the largest interaction energy suggesting that a more accurate method is required. This observation is confirmed by the SAPT(RHF) and supermolecular DFT results which correctly score ligand-5 as the most potent in this set.

In the lower panel of Fig. 5(d) we plot the first order interaction energy difference, Δ*E*^(1)^_int_ computed through SAPT(RHF), SAPT(CASCI) and SAPT(VQE) all computed using a monomer-centered basis. The first thing we see is the excellent agreement between SAPT(CASCI) and SAPT(VQE) for each ligand considered. Note that we used the 1-muCJ ansatz for the VQE computations which offers further evidence of the error reduction capabilities of SAPT. Apart from this excellent agreement we also see that there is little difference between SAPT(RHF) and SAPT(CASCI). We found that the maximum difference between SAPT(CASCI) and SAPT(RHF) was roughly 1–2 kcal mol^−1^, however this error nearly cancels when looking at the Δ*E*_int_ and is thus not visible on the scale of the plot. Ultimately this is not surprising given that the problem was found to be not strongly correlated.

Finally, we see that first order SAPT is not accurate enough to correctly score the respective ligands with the missing induction and dispersion components proving critical to achieving this. Indeed the first order interaction energy for ligand-5 is positive and thus unbound at this level of theory. In particular, from the full SAPT^(2)^(RHF) energy decomposition (see Supplementary Information) we find that the induction components for ligand-4 and ligand-5 are similar in magnitude while the dispersion component for ligand-5 is roughly 1.4 times that of ligand-4. This large dispersion energy ensures that ligand-5 has the larger (absolute) interaction energy. Nevertheless, we see that SAPT(VQE) can potentially tackle industrially relevant drug design problems, particularly in cases where it is not known *a priori* how strongly correlated the ground state is.

## III. Conclusion

We described the theory and implementation of SAPT on a NISQ-era quantum computer, focusing on the efficient implementation and classical workflows necessary to tackle industrially relevant problems in drug design. We derived in detail the equations necessary for an active space formulation of SAPT(VQE) that requires only the one- and two-particle reduced density matrices measured on a quantum computer. The SAPT(VQE) components are computed as an efficient classical post-processing step. This classical post-processing step, written in terms of optimized quantum chemical primitives, was shown capable of simulating systems with hundreds of atoms and potentially hundreds of basis functions in the active space.

Beyond an efficient classical implementation, we found (through ideal VQE simulations) that SAPT naturally reduces the error incurred by approximately solving the Schrödinger equation on a quantum computer, which we attribute to the theory directly computing energy differences. This fact coupled with a monomer basis formulation will substantially reduce the resource requirements for computing binding energies of large protein–ligand interactions on NISQ-era quantum computers. In particular, sub-kcal mol^−1^ accuracy in the energy components for the electrostatic and exchange energies can be computed using coarse VQE wavefunctions, which otherwise exhibit gross errors (>10 kcal mol^−1^) in the total energy. Given the practical challenges associated with optimizing VQE wavefunctions on current hardware we believe SAPT may help to extend the scope of the method due to the apparent ability to use low depth VQE ansatzes like 1-uCJ. SAPT(VQE) appears to offer, then, a reduction in the quantum resources required compared to a simple supermolecular VQE for the computation of protein–ligand interaction energies. This reduction in qubit count and circuit depth naturally yields a lower precision requirement on the quantum circuit and thus has the potential to enable the simulation of larger systems on NISQ hardware than what is possible with supermolecular VQE implementations.

In the future, it will be critical to determine how robust SAPT(VQE) is to noise channels either through modeling or real hardware experiments. Another important question will be how to reduce the measurement overhead of accumulating the one- and two-particle reduced density matrices, a tentative solution to which is sketched in Appendix B. A natural extension will be to determine the second-order SAPT terms that would allow for accurate interaction energies and induction and dispersion energy components to be computed. Other interesting questions are how to increase the quantitative accuracy of the method by including correlation out of the active space^18,59^ and to gather more challenging drug–protein systems potentially containing multiple metal centers.

## IV. Appendix A: full active space SAPT exchange expressions

In this appendix we give full expressions for the remaining exchange energy contributions (*T*_3_ and *T*_4_) not given in Section ID. Note that we will often perform optimizations similar to those provided by eqn (41) with the general strategy to be the removal of core orbital indices from the generalized ERI expressions through the formation of appropriate intermediate matrices.

*T*_3_ is very similar to *T*_2_ and we haveA1
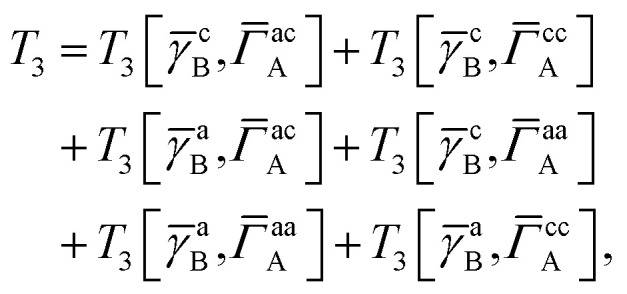
whereA2
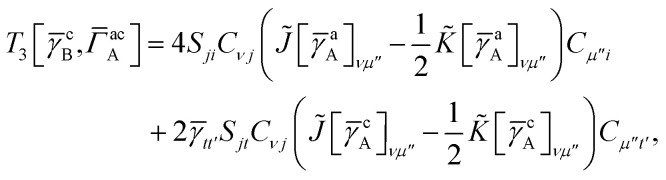
A3
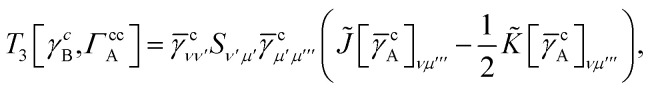
A4
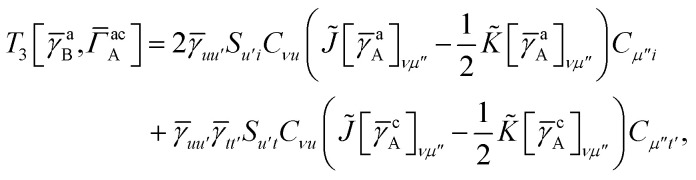
A5
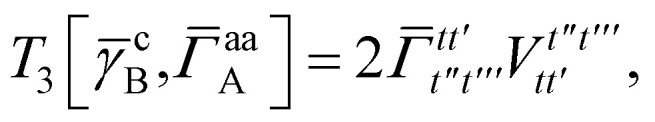
where as in eqn (41) we have definedA6
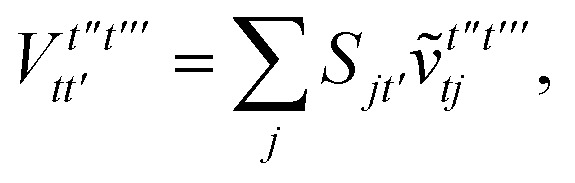
and, lastly,A7
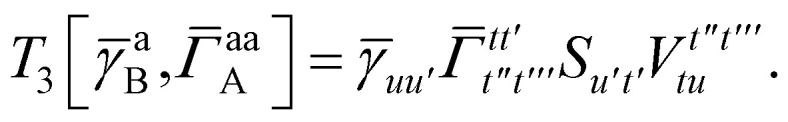


For *T*_4_ there are sixteen terms in total. First we haveA8
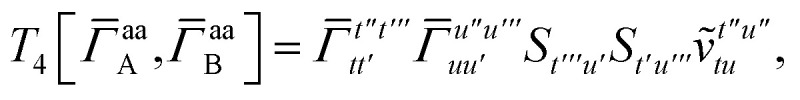
which can't be simplified further. Next we haveA9
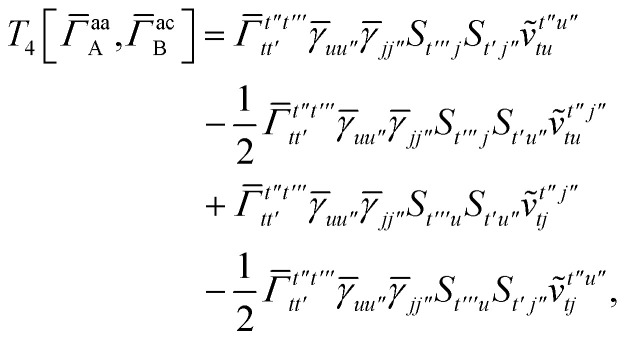
which can be written asA10
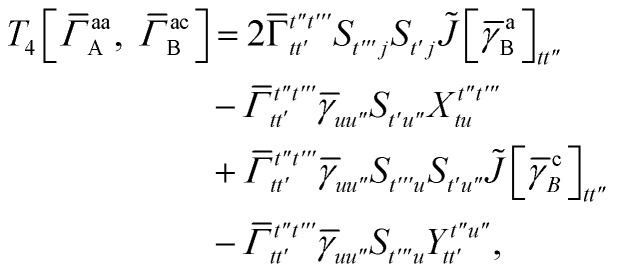
whereA11
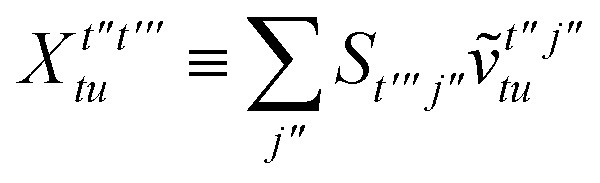
A12
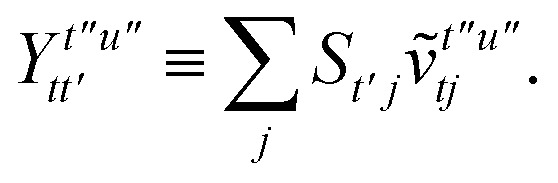


SimilarlyA13
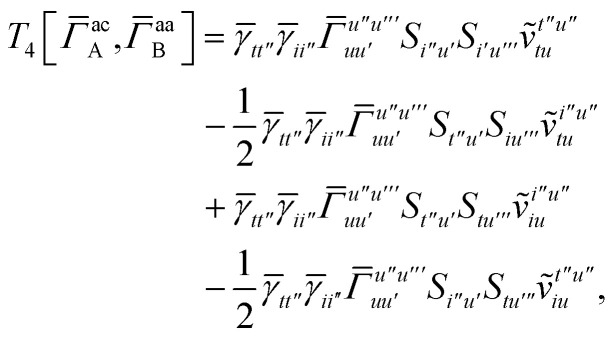
which can be written asA14
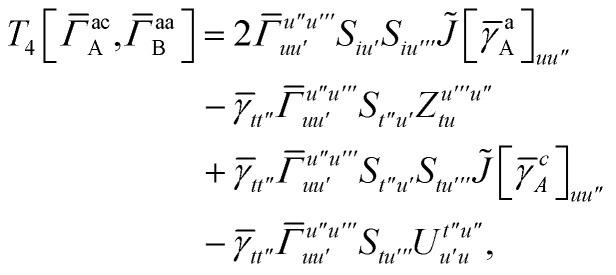
whereA15
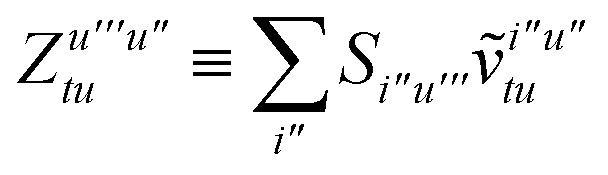
A16
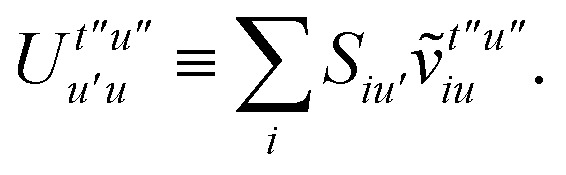


NextA17
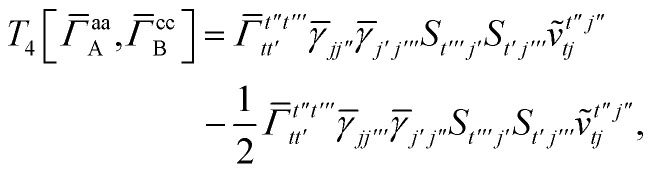
which can be written asA18
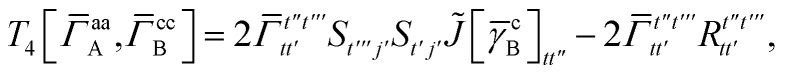
whereA19
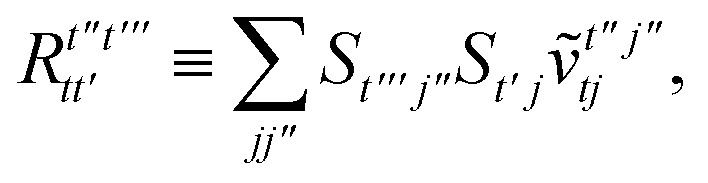
and similarlyA20
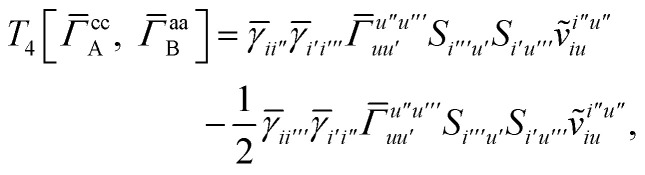
which can be written asA21
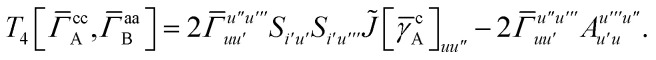
A22
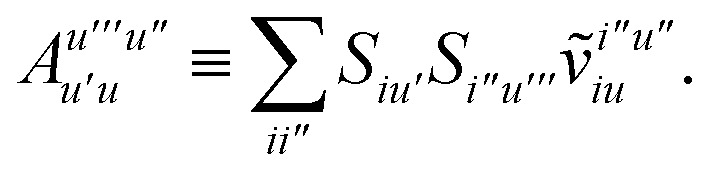


For the AC–AC contribution we get sixteen termsA23
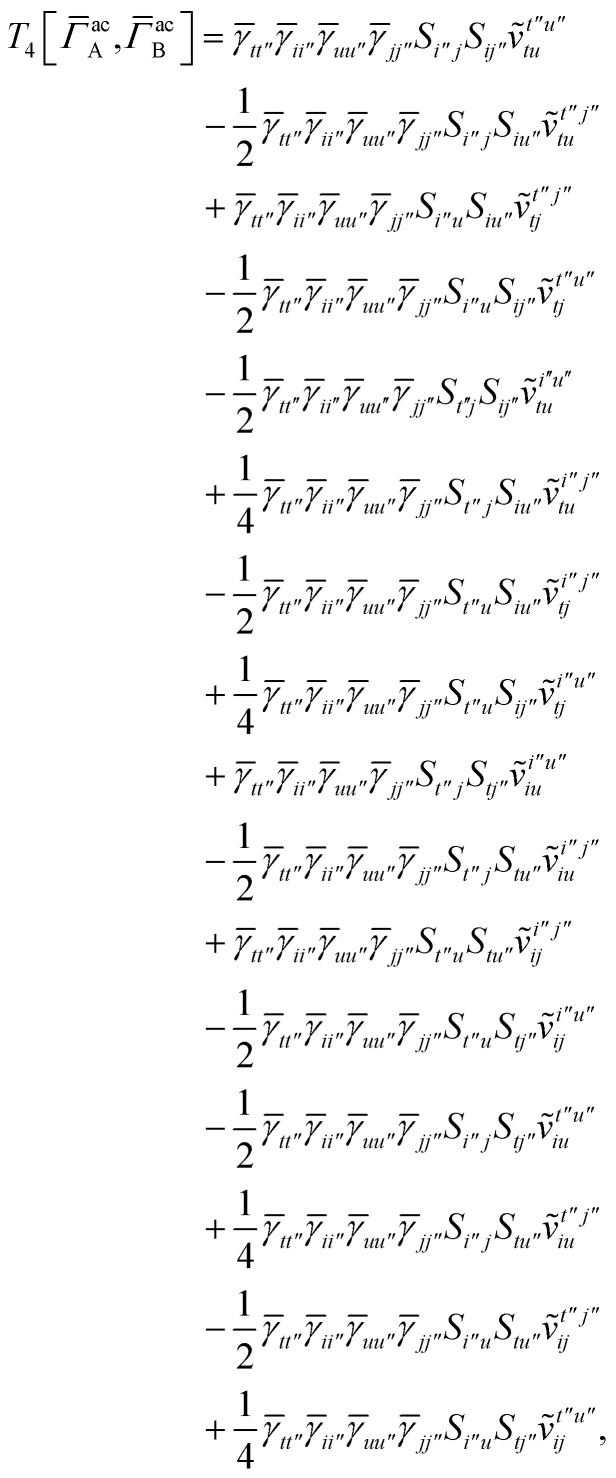
which can be simplified toA24
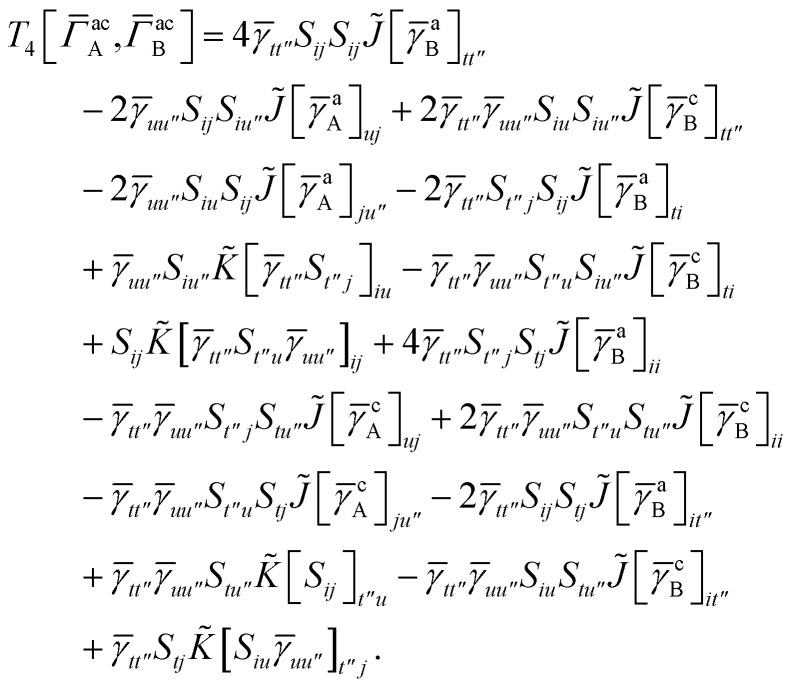


Next we haveA25
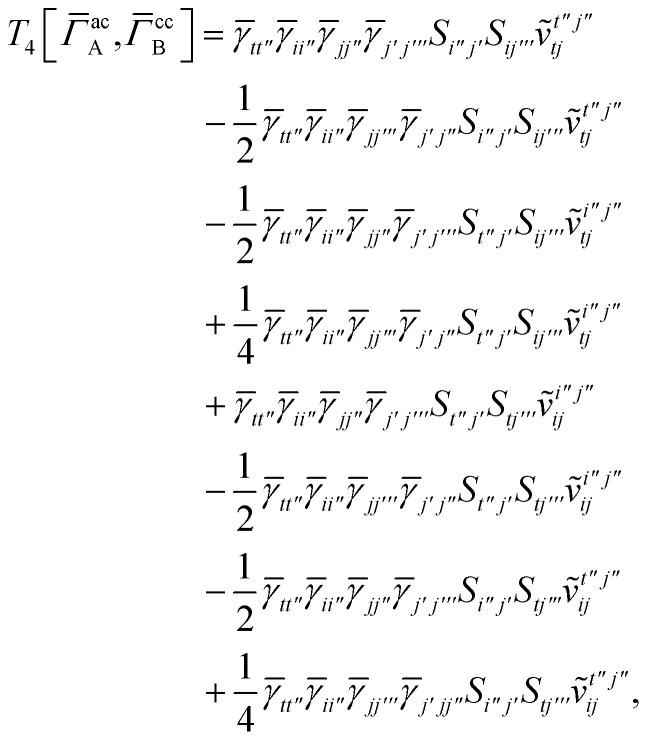
which can be written asA26
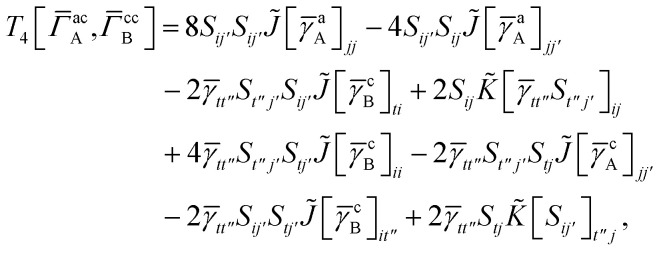
and similarlyA27
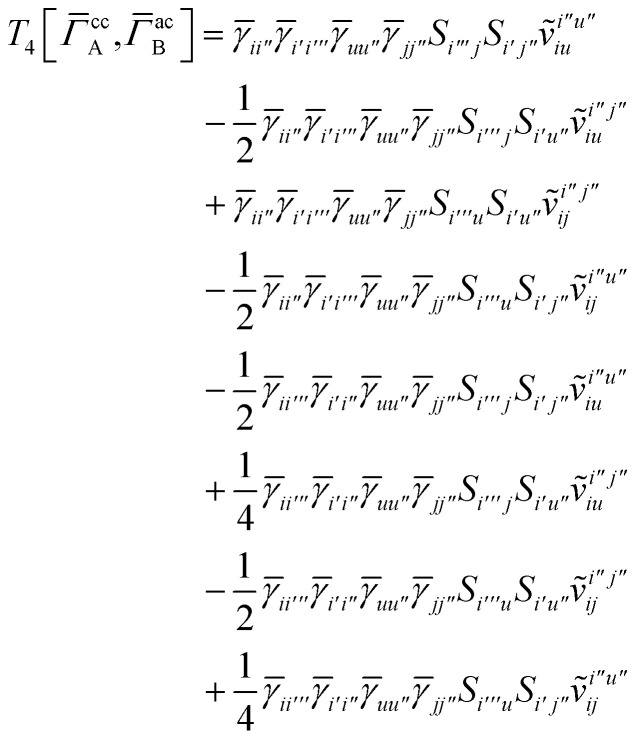
which can be written asA28
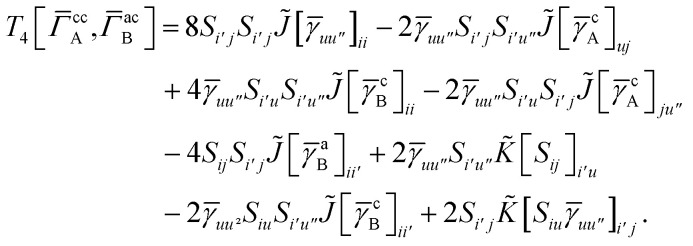


Finally, we haveA29
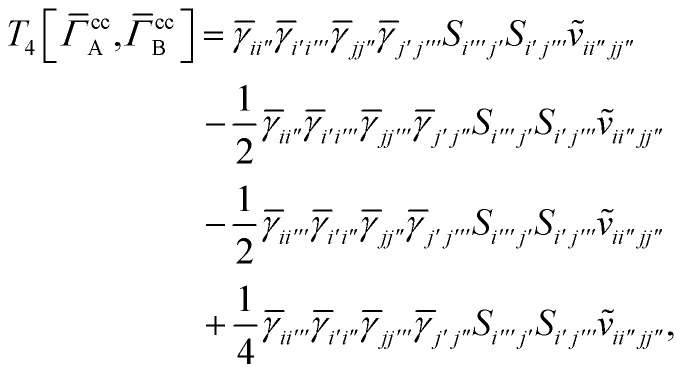
which can be written in terms of *J̃* and *K̃* matrices as was done for the SAPT(HF).

## V. Appendix B: quantum–classical optimization for electrostatic energy

When only monomer A is defined to be quantum, an interesting alternative approach exists to evaluating the one-particle density matrix on the quantum computer. We may first classically form the contributions from the nuclei of monomer A,B1
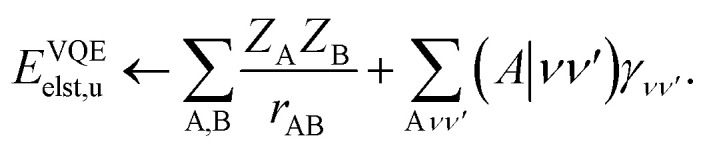


We then classically form the image of the electrostatic potential of monomer B in the atomic orbital basis of monomer A as,B2
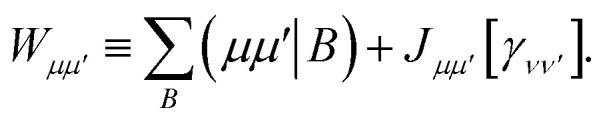


We can form the monomer A core ↔ monomer B contributions classically as,B3
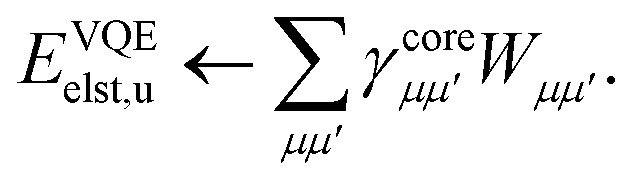


We can then classically form the image of the electrostatic potential of monomer B in the active space molecular orbital basis of monomer A as,B4
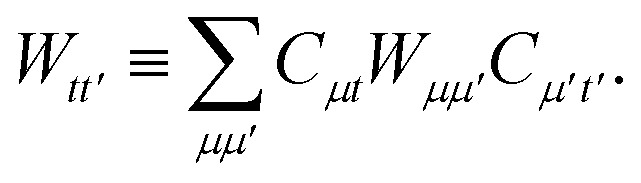


We can then diagonalize the *W*_*tt*′_ operator to form,B5
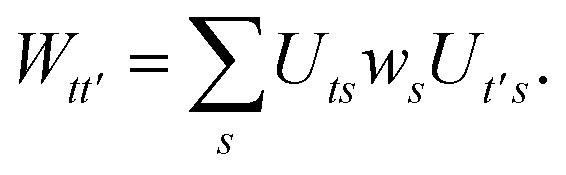
where *U*_ts_ is 
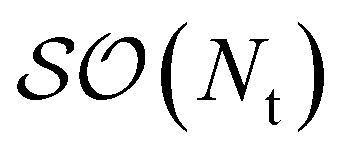
. In this “electrostatic potential natural orbital basis” the remaining monomer A active ↔ monomer B contributions can be evaluated by a single commuting group of simultaneous *Z*-basis measurements,B6
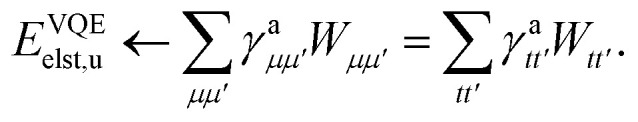
B7
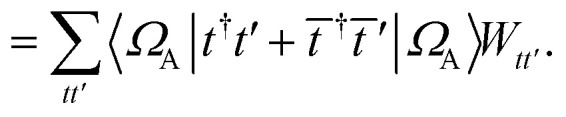
B8
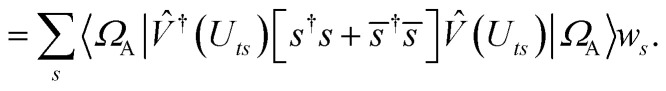
B9
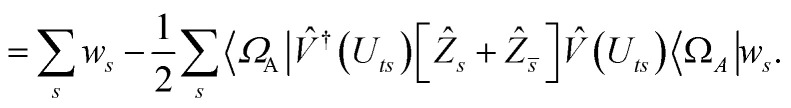


This is in contrast to a naive implementation which would require 
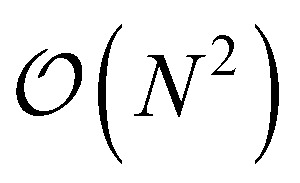
 circuit evaluations or an optimal method of *N*/2 circuits evaluations.^41^ Similar ideas can be explored for the exchange expression. Given that its structure mirrors that of a total energy evaluation (a 4-index tensor contracted with the two-particle reduced density matrix) it seems possible that a double factorization^45,46^ approach may be possible. These ideas will be explored at a later date.

## Data availability

Molecular geometries, raw data and additional calculations are available in the supporting information.

## Author contributions

R. M. P conceived and directed this project. R. M. P. and F. D. M derived and implemented the SAPT(VQE) method. F. D. M. ran and analyzed the SAPT(VQE) simulations. T. F. prepared the KDM5A structures, assisted with their visualization and helped analyze the data. N. M. optimized the KDM5A geometries and computed the DFT binding energies. A. R. W., M. D., E. K., R. S., N. M. and M. S. critiqued and helped improve SAPT(VQE). All authors discussed the results and wrote the manuscript.

## Conflicts of interest

F. D. M., R. M. P., and A. R. W. own stock/options in QC Ware Corp.

## Supplementary Material

SC-013-D1SC05691C-s001

SC-013-D1SC05691C-s002

SC-013-D1SC05691C-s003

SC-013-D1SC05691C-s004

SC-013-D1SC05691C-s005

SC-013-D1SC05691C-s006
