# Supplementary material for: Towards the simulation of large scale protein–ligand interactions on NISQ-era quantum computers
Source: Chem Sci. 2022 Jan 17;13(11):3094–108. doi: 10.1039/d1sc05691c (PMC8926290; doi:10.1039/d1sc05691c)
Supplement: SC-013-D1SC05691C-s006 [file SC-013-D1SC05691C-s006.pdf]

Electronic Supplementary Material (ESI) for Chemical Science.
This journal is © The Royal Society of Chemistry 2022

Supplementary Information for Towards the Simulating Large Scale Protein-Ligand Interactions on NISQ-era Quantum Computers

Fionn D. Malone, Robert M. Parrish ,* and Alicia R. Welden
QC Ware Corporation, Palo Alto, CA, 94301, USA

Thomas Fox
Medicinal Chemistry, Boehringer Ingelheim Pharma GmbH & Co. KG,
Birkendorfer Straße 65, 88397 Biberach an der Riß, Germany

Matthias Degroote, Elica Kyoseva, Nikolaj Moll ,† Raffaele Santagati, and Michael Streif
Quantum Lab, Boehringer Ingelheim, 55218 Ingelheim am Rhein, Germany

I. DATA FOR BENZENE–P-BENZYNE DIMER

In Table I and Table II we present results for the benzene–p-benzyne dimer considered in the main text. All calculations were performed in the (monomer-centered) cc-pVDZ basis set and a gradient threshold of 1×10^{-6} for the L-BFGS-B solver in scipy. For comparison in Fig. 1 we compare the SAPT(RHF) energy components in the monomer-centered and dimer-centered basis sets. We found that in the monomer-centered basis set the exchange energy became a small negative number which is likely a numerical artifact but was consistent across different codes. Finally, in Fig. 2 we plot the convergence of the errors in the one- and two-particle density matrices as a function of the circuit repetition factor k in the k-muCJ ansatz.

R (Å)	SAPT(RHF)	SAPT(CASCI)	SAPT(1-uCJ)	SAPT(5-uCJ)
3.950000	-0.017566	-0.018136	-0.018212	-0.018162
4.131818	-0.008560	-0.009209	-0.009319	-0.009251
4.313636	-0.003534	-0.004184	-0.004303	-0.004138
4.495455	-0.000865	-0.001477	-0.001594	-0.001448
4.677273	0.000461	-0.000097	-0.000205	-0.000129
4.859091	0.001052	0.000552	0.000454	0.000575
5.040909	0.001255	0.000813	0.000726	0.000796
5.222727	0.001267	0.000877	0.000800	0.000895
5.404545	0.001191	0.000848	0.000780	0.000835
5.586364	0.001082	0.000779	0.000720	0.000768
5.768182	0.000966	0.000699	0.000647	0.000683
5.950000	0.000855	0.000620	0.000573	0.000636

TABLE I: First order E_{elst} for benzene–p-benzyne dimer as a function of the center-to-center distance R (in angstrom) for different levels of SAPT. Energies are in Hartree.

II. DATA FOR H_2O

In Tables III to V we present RHF, CASCI and VQE SAPT results for the water dimer considered as a function of the stretched OH bond length in one of the monomers. We also provide for reference the total energy of the isolated water molecule undergoing the symmetric stretch. All calculations were performed in the (monomer-centered) 6-31g basis set and a gradient threshold of 1×10^{-6} for the L-BFGS-B solver in scipy or with a maximum iteration of 1000 whichever occurred first. In cases when the maximum iteration was reached first we found the norm of the gradient to be $\approx 1 \times 10^{-5}$. In order to obtain a relatively smooth potential energy curve we started from the stretched geometry with randomly chosen parameters. We then used these optimized parameters for the next more compressed geometry and so on. To check the dependence of the energies on the quality of the VQE solution we plot in Fig. 4 the VQE

* rob.parrish@qcware.com
† nikolaj.moll@boehringer-ingelheim.com

R	SAPT(RHF)	SAPT(CASCI)	SAPT(1-uCJ)	SAPT(5-uCJ)
3.950000	0.044729	0.046125	0.046124	0.046149
4.131818	0.023684	0.024907	0.024942	0.025003
4.313636	0.011746	0.012730	0.012727	0.012718
4.495455	0.005388	0.006068	0.006079	0.006039
4.677273	0.002230	0.002668	0.002682	0.002674
4.859091	0.000787	0.001053	0.001064	0.001045
5.040909	0.000196	0.000345	0.000357	0.000347
5.222727	-0.000008	0.000074	0.000080	0.000074
5.404545	-0.000055	-0.000012	-0.000009	-0.000012
5.586364	-0.000050	-0.000028	-0.000027	-0.000028
5.768182	-0.000034	-0.000023	-0.000022	-0.000023
5.950000	-0.000020	-0.000015	-0.000014	-0.000015

TABLE II: First order E_{exch} for benzene–p-benzyne dimer as a function of the center-to-center distance R (in angstrom) for different levels of SAPT. Energies are in Hartree.

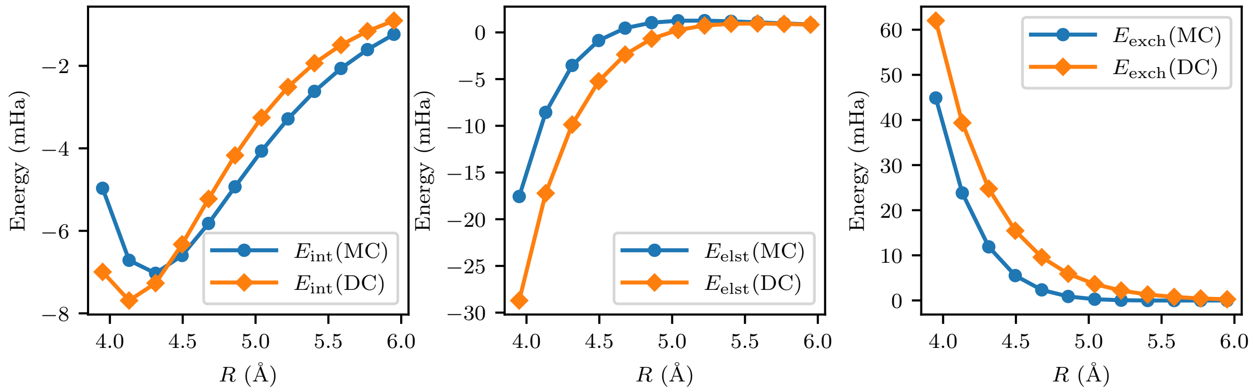

FIG. 1: Comparison between monomer-centered (MC) and dimer-centered(DC) SAPT(RHF) energy components for the benzene–p-benzyne dimer. Note unlike other calculations presented in this work, these results were computed using the density-fitting version of SAPT [1, 2] implemented in the psi4 quantum chemistry package [3].

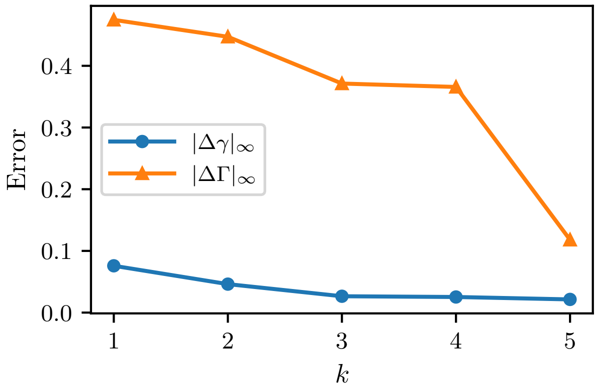

FIG. 2: Error in the one and two particle density matrix as a function of the circuit repetition factor k for the p-benzyne molecule. Here $|\Delta\gamma|_\infty = |\gamma_{\text{CASCI}} - \gamma_{k-\text{muCJ}}|_\infty$ and $|\Delta\Gamma|_\infty = |\Gamma_{\text{CASCI}} - \Gamma_{k-\text{muCJ}}|_\infty$ where $|x|_\infty = \max_i \sum_j |x_{ij}|$ is the infinity norm. Due to some conventional differences between lightspeed and our VQE code we used PySCF to compute the reference CASCI two-particle density matrices[4].

total energy, electrostatic and exchange energies as a function of the gradient threshold. We see that the errors are quite well converged around 1×10^{-3} and the SAPT components are quite insensitive to how well optimized the wavefunction is. In Fig. 5 we compare the SAPT(RHF), SAPT(CASCI) and SAPT(CASSCF) to test the importance

```
C    -1.3940633    0.0000000   -2.0000000
C    -0.6970468    1.2072378   -2.0000000
C     0.6970468    1.2072378   -2.0000000
C     1.3940633    0.0000000   -2.0000000
C     0.6970468   -1.2072378   -2.0000000
C    -0.6970468   -1.2072378   -2.0000000
H    -2.4753995    0.0000000   -2.0000000
H    -1.2382321    2.1435655   -2.0000000
H     1.2382321    2.1435655   -2.0000000
H     2.4753995    0.0000000   -2.0000000
H     1.2382321   -2.1435655   -2.0000000
H    -1.2382321   -2.1435655   -2.0000000
--
C     0.0000000    0.0000000    1.0590353
C     0.0000000   -1.2060084    1.7576742
C     0.0000000   -1.2071767    3.1515905
C     0.0000000    0.0000000    3.8485751
C     0.0000000    1.2071767    3.1515905
C     0.0000000    1.2060084    1.7576742
H     0.0000000   -2.1416387    1.2144217
H     0.0000000   -2.1435657    3.6929953
H     0.0000000    2.1435657    3.6929953
H     0.0000000    2.1416387    1.2144217
```

FIG. 3: Coordinates for benzene and p-benzyne dimer given at a center-to-center distance of $R = 4.45$ Å.

of orbital optimization for SAPT(VQE). We see that qualitatively SAPT(CASCI) captures the correct behaviour in the stretched geometry but there is some quantitative difference. Finally in Fig. 6 we provide the xyz coordinates for the water dimer at $R_{\mathrm{OH}} = 0.2397$ Å.

R	E_{elst}	E_{exch}	E_{RHF}
0.2397	-0.009707	0.005482	-63.545181
0.5035	-0.014737	0.006620	-74.456288
0.7672	-0.016340	0.006510	-75.869444
1.0309	-0.016566	0.006208	-75.973944
1.2946	-0.015416	0.005953	-75.865638
1.5583	-0.013711	0.005811	-75.738337
1.8220	-0.012270	0.005758	-75.624465
2.0857	-0.011754	0.005843	-75.528585
2.3494	-0.010643	0.005758	-75.449977
2.6132	-0.010370	0.005778	-75.386652

TABLE III: RHF electrostatic, exchange energies for the water dimer system as a function of the OH bond length in monomer 2 and the total RHF energy of monomer 2.

R	E_{elst}	E_{exch}
0.239739	-0.009756	0.005559
0.503451	-0.014742	0.006671
0.767163	-0.016210	0.006515
1.030876	-0.015636	0.006245
1.294588	-0.012944	0.006107
1.558300	-0.009807	0.006103
1.822013	-0.007061	0.006195
2.085725	-0.005088	0.006305
2.349438	-0.004132	0.006369
2.613150	-0.003750	0.006391

TABLE IV: CASCI electrostatic and exchange energies for the water dimer system as a function of the OH bond length in monomer 2.

R	k	E_{elst}	E_{exch}	E_{VQE}	ΔE
0.2397	1	-0.009756	0.005559	-63.549642	-0.000029
0.5035	1	-0.014745	0.006672	-74.463219	-0.000073
0.7672	1	-0.016247	0.006524	-75.873483	-0.000181
1.0309	1	-0.015858	0.006244	-75.993030	-0.001175
1.2946	1	-0.013001	0.006113	-75.921709	-0.000478
1.5583	1	-0.009738	0.006102	-75.840780	-0.005728
1.8220	1	-0.007066	0.006188	-75.785824	-0.006303
2.0857	1	-0.005266	0.006288	-75.752021	-0.009017
2.3494	1	-0.004303	0.006348	-75.736872	-0.012412
2.6132	1	-0.003858	0.006377	-75.729624	-0.015426
0.2397	2	-0.009756	0.005556	-63.549658	-0.000013
0.5035	2	-0.014737	0.006671	-74.463238	-0.000054
0.7672	2	-0.016206	0.006514	-75.873605	-0.000059
1.0309	2	-0.015625	0.006240	-75.993936	-0.000268
1.2946	2	-0.013014	0.006107	-75.921894	-0.000294
1.5583	2	-0.009835	0.006102	-75.846398	-0.000111
1.8220	2	-0.007072	0.006192	-75.792062	-0.000065
2.0857	2	-0.005091	0.006305	-75.760956	-0.000082
2.3494	2	-0.004135	0.006369	-75.749237	-0.000047
2.6132	2	-0.003747	0.006390	-75.745000	-0.000050
0.2397	4	-0.009756	0.005560	-63.549668	-0.000003
0.5035	4	-0.014742	0.006670	-74.463277	-0.000015
0.7672	4	-0.016210	0.006515	-75.873653	-0.000011
1.0309	4	-0.015638	0.006245	-75.994190	-0.000015
1.2946	4	-0.012945	0.006107	-75.922173	-0.000014
1.5583	4	-0.009807	0.006103	-75.846502	-0.000006
1.8220	4	-0.007060	0.006194	-75.792118	-0.000009
2.0857	4	-0.005090	0.006305	-75.761019	-0.000019
2.3494	4	-0.004130	0.006368	-75.749260	-0.000024
2.6132	4	-0.003783	0.006400	-75.745017	-0.000034

TABLE V: VQE electrostatic, exchange energies for the water dimer system as a function of the OH bond length in monomer 2 for different values of the k parameter in the k-uCJ ansatz. Also given is the total VQE energy of monomer 2 and the deviation of VQE from CASCI.

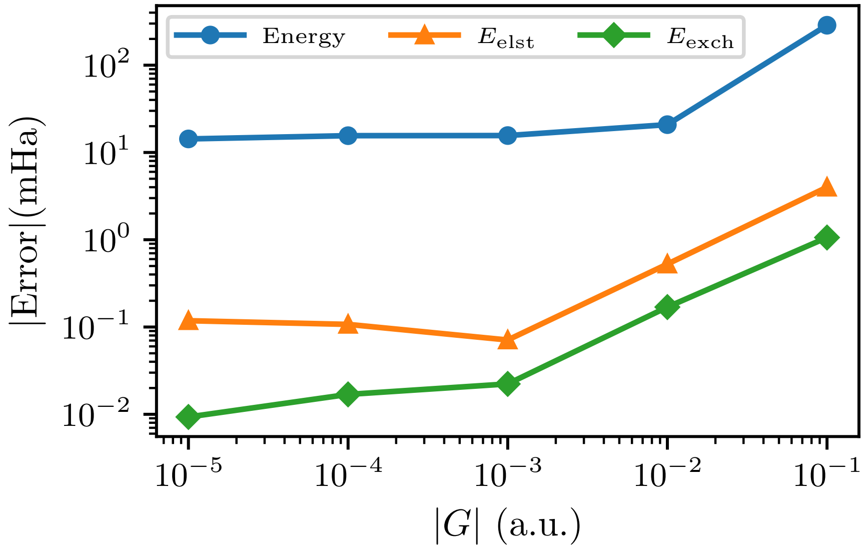

FIG. 4: Convergence of errors (relative to CASCI) for the total energy, electrostatic and exchange energies when using the modified 1-uCJ ansatz as a function of the gradient threshold for the L-BFGS-B algorithm implemented in scipy (gtol). To obtain the data we first initialised the parameters randomly and fed the output parameters from one BFGS optimization at a larger threshold into the next optimization step with a smaller threshold.

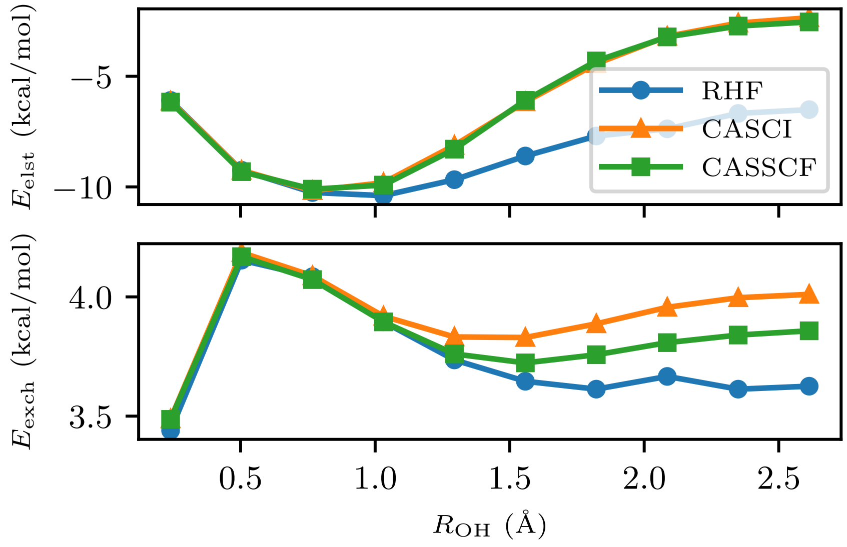

FIG. 5: Comparison between SAPT(RHF), SAPT(CASCI) and SAPT(CASSCF) electrostatic and exchange energies as a function one of the monomers OH bond length.

```
O  -1.551007  -0.114520   0.000000
H  -1.934259   0.762503   0.000000
H  -0.599677   0.040712   0.000000
--
O   1.350625   0.111469   0.000000
H   1.433068  -0.009834  -0.189640
H   1.433068  -0.009834   0.189640
```

FIG. 6: Coordinates for H_2O dimer given with $R_{\mathrm{OH}} = 0.2397$ in the second monomer.

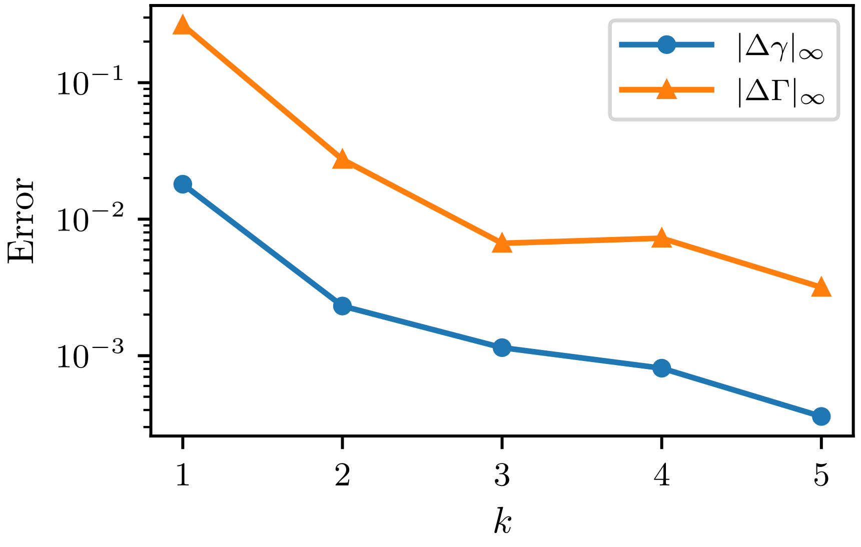

FIG. 7: Error in the one and two particle density matrix as a function of the circuit repetition factor k for the H_2O molecule with $R_{\mathrm{OH}} = 1.438$ Å. Note the $k = 1$ VQE energy differs from CASCI by 3.2 kcal/mol at this OH bond length. Here $|\Delta\gamma|_\infty = |\gamma_{\mathrm{CASCI}} - \gamma_{k-\mathrm{muCJ}}|_\infty$ and $|\Delta\Gamma|_\infty = |\Gamma_{\mathrm{CASCI}} - \Gamma_{k-\mathrm{muCJ}}|_\infty$ where $|x|_\infty = \max_i \sum_j |x_{ij}|$ is the infinity norm. Due to some conventional differences between lightspeed and our VQE code we used PySCF to compute the reference CASCI two-particle density matrices[4].

III. DATA AND INFORMATION FOR KDM5A SYSTEM

Below we provide further information about the preparation of the KDM5A structure studied in this work as well as raw energies. Note we provide the xyz files for these structures as attached supplemental files instead of reproducing their coordinates in this pdf.

A. Cutout Preparation for KDM5A

To make the protein tractable, we used a model system of the binding site which was cut out from the full binding domain of KDM5A. Starting from the Xray structure with ligand 5 cocrystallized (pdb code 6bh4), in the molecular modeling program MOE [5] the following preparation steps were performed: (1) remove all other small molecule ligands outside the binding site, keeping all Xray waters. (2) add missing side chains with the *Prepare Structure* module of MOE. (3) Replace the Mn2+ in the Xray structure with Fe3+. (4) bridge a one-amino-acid break in the protein chain by manually adding and minimizing an alanine residue A357 and cap the protein ends of a missing 9-residue loop with an ACE and NME, respectively. (5) add hydrogens with the *Protonate3D* module of MOE. (6) with the MOE module *Quickprep* perform a series of tethered minimizations with decreasing tether weights of 100, 50, 10, 5 kcal/mol, keeping all atoms further than 8 A from the ligand fixed, using a maximal gradient of 0.5 kcal/mol $Å^{-2}$, and a flat well potential ± 0.25 Åfrom the initial atom positions. With the protein structure thus prepared, we removed all protein and water residues further than 4.5 A away from the ligand and capped the C- and N-termini of the fragmented protein with NME or ACE. This was followed by additional manual pruning by removing amino acid residues, sidechain or backbone atoms, do not interact with the ligand or the Fe ion. Only three water molecules were retained – two interacting with the Fe ion, and one interacting with the southern nitrogen atom of the 6-membered aromatic ring of the ligand. The resulting protein model system consisted of 163 atoms, 88 of which were heavy atoms. Based on the Xray structure of ligand 5, we modelled the binding modes of ligands 4, 8, and 9, and 12. For ligands 8 and 9, a tethered minimization was necessary to resolve a few close contacts between ligand and protein; for ligand 12 with its significantly different hydrogen-bond characteristics, we re-ran *Protonate 3D* to optimize the hydrogen-bond network around the ligand.

B. Energy Data

System	RHF	VQE (1-uCJ)	CASCI
Protein	-10829.177918	-10829.204970	-10829.220470
Ligand 4	-598.793891	N/A	N/A
Protein	-10829.175107	-10829.201205	-10829.217807
Ligand 5	-715.843329	N/A	N/A
Protein	-10829.238106	-10829.278175	-10829.282006
Ligand 8	-714.693949	N/A	N/A
Protein	-10829.227796	-10829.253366	-10829.269886
Ligand 9	-714.714317	N/A	N/A
Protein	-10829.182313	-10829.206071	-10829.224031
Ligand 12	-692.814843	N/A	N/A

TABLE VI: RHF, VQE and CASCI total energies for the relaxed protein and ligands considered in this work. Energies are in Hartree atomic units.

Compound	$E_{\text{elst}}^{\text{RHF}}$	$E_{\text{exch}}^{\text{RHF}}$	$E_{\text{elst}}^{\text{CASCI}}$	$E_{\text{exch}}^{\text{CASCI}}$	$E_{\text{elst}}^{\text{VQE}}$	$E_{\text{exch}}^{\text{VQE}}$
4	-0.179402	0.129074	-0.180031	0.129294	-0.179813	0.129323
5	-0.177596	0.134383	-0.178216	0.134567	-0.178140	0.134560
8	-0.170699	0.137911	-0.171302	0.138117	-0.171266	0.138075
9	-0.155808	0.139853	-0.157447	0.140533	-0.156897	0.140413
12	-0.164345	0.138107	-0.164988	0.138316	-0.164799	0.138228

TABLE VII: RHF, VQE and CASCI first order SAPT contributions for the relaxed protein and ligands considered in this work. Energies are in Hartree atomic units. Note monomer centered basis sets and the S^2 approximation for exchange were employed.

Component	Compound 4	Compound 5
E_{elst}	-204.242	-208.646
E_{exch}	203.780	226.393
E_{ind}	-66.821	-68.796
E_{dis}	-99.107	-138.142
$E^{(1)}$(SAPT)	-0.462	17.747
$E^{(2)}$(SAPT)	-165.928	-206.938
E_{int}(SAPT)	-166.390	-189.192

TABLE VIII: Breakdown of SAPT(RHF) contributions to total interaction energy. Energies are in milli-Hartree. Note a *dimer* centered basis set and the S^∞ exchange were employed which should be contrasted to other calculations presented in this work. The SAPT(RHF) method employed here is the 'SAPT(0.5)' method of Parrish *et al.* which approximates the dispersion energy[6].

[1] E. G. Hohenstein and C. D. Sherrill, Density fitting and Cholesky decomposition approximations in symmetry-adapted perturbation theory: Implementation and application to probe the nature of π-π interactions in linear acenes, J. Chem. Phys. **132**, 184111 (2010).

[2] E. G. Hohenstein, R. M. Parrish, C. D. Sherrill, J. M. Turney, and H. F. S. III, Large-scale symmetry-adapted perturbation theory computations via density fitting and Laplace transformation techniques: Investigating the fundamental forces of DNA-intercalator interactions, J. Chem. Phys. **135**, 174107 (2011).

[3] J. M. Turney, A. C. Simmonett, R. M. Parrish, E. G. Hohenstein, F. A. Evangelista, J. T. Fermann, B. J. Mintz, L. A. Burns, J. J. Wilke, M. L. Abrams, N. J. Russ, M. L. Leininger, C. L. Janssen, E. T. Seidl, W. D. Allen, H. F. Schaefer, R. A. King, E. F. Valeev, C. D. Sherrill, and T. D. Crawford, Psi4: an open-source ab initio electronic structure program, Wiley Interdiscip. Rev. Comput. Mol. Sci. **2**, 556 (2012).

[4] Q. Sun, T. C. Berkelbach, N. S. Blunt, G. H. Booth, S. Guo, Z. Li, J. Liu, J. D. McClain, E. R. Sayfutyarova, S. Sharma, S. Wouters, and G. K.-L. Chan, Pyscf: the python-based simulations of chemistry framework, Wiley Interdiscip. Rev. Comput. Mol. Sci. **8**, e1340 (2018).

[5] S. Vilar, G. Cozza, and S. Moro, Medicinal Chemistry and the Molecular Operating Environment (MOE): Application of QSAR and Molecular Docking to Drug Discovery, Curr. Top. Med. Chem. **8**, 1555 (2008).

[6] R. M. Parrish, K. C. Thompson, and T. J. Martínez, Large-Scale Functional Group Symmetry-Adapted Perturbation Theory on Graphical Processing Units, J. Chem. Theory Comput. **14**, 1737 (2018).
